# GiantHost: a domain-adaptive and uncertainty-aware framework for giant virus host prediction

**DOI:** 10.1093/bioinformatics/btag498

**Published:** 2026-08-21

**Authors:** Fuchuan Qu, Guowei Chen, Yanni Sun

**Affiliations:** Department of Electrical Engineering, City University of Hong Kong, Hong Kong (SAR), 999077, China; Department of Electrical Engineering, City University of Hong Kong, Hong Kong (SAR), 999077, China; Department of Electrical Engineering, City University of Hong Kong, Hong Kong (SAR), 999077, China

## Abstract

**Motivation:**

Nucleocytoplasmic large DNA viruses (NCLDVs) play crucial roles in global ecosystems. Although metagenomics has vastly accelerated the discovery of novel NCLDVs, predicting their hosts from fragmented contigs remains a critical bottleneck, with no dedicated end-to-end computational tools currently available. Addressing this gap requires overcoming three fundamental challenges: the extreme scarcity of labeled reference genomes, the severe domain shift between laboratory isolates and diverse environmental metagenomes, and the inability of traditional deterministic models to quantify prediction uncertainty—a crucial requirement for reliable ecological profiling where novel, divergent viruses are prevalent.

**Results:**

We present GiantHost, the first NCLDV host prediction tool with domain adaptation and uncertainlty awareness. GiantHost employs a dual-tower neural network to integrate dense genome traits and sparse GVOG profiles, allowing better integration of heterogeneous features. To overcome label scarcity and domain shift, we leverage 1400 environmental viral genomes (GVMAGs) via semi-supervised multi-task learning and Domain Adversarial Neural Networks (DANN), effectively bridging the distributional gap between RefSeq and environmental data. Additionally, GiantHost incorporates Conformal Prediction (CP) to output statistically guaranteed prediction sets rather than overconfident single labels. Evaluated under rigorous genome-level cross-validation, GiantHost demonstrates robust predictive power. Applied to the Tara Ocean dataset, GiantHost successfully captured the vertical stratification of NCLDV hosts—revealing a depth-dependent decline of phytoplankton-infecting viruses and a relative enrichment of Amoebozoa-infecting viruses in the mesopelagic zone.

**Availability:**

The source code of GiantHost is available via: https://github.com/FuchuanQu/GiantHost.

## 1 Introduction

Giant viruses, or nucleocytoplasmic large DNA viruses (NCLDVs), are characterized by exceptionally large double-stranded DNA genomes (up to 2.5 Mbp) and complex functional repertoires (Yi *et al.* 2024). They are increasingly recognized as pervasive and influential members of global aquatic ecosystems, capable of reprogramming host metabolism during infection and significantly impacting biogeochemical cycling (Moniruzzaman *et al.* 2020). The advent of large-scale metagenomic surveys has enabled the recovery of vast numbers of NCLDV sequences directly from environmental samples (Moniruzzaman *et al.* 2020; Schulz *et al.* 2020). However, understanding the ecological roles of these newly discovered viruses requires identifying their hosts. While metagenomic sequencing generates abundant viral contigs, computationally predicting their eukaryotic hosts remains a major challenge.

Host prediction can be formulated as a link-prediction or multi-classification problem, depending on whether the host-derived features are explicitly utilized. Link prediction predicts whether a specific virus interacts with a specific host (Galiez *et al.* 2017, Shang and Sun 2022). For instance, CHERRY formulates this as a knowledge graph link prediction task (Shang and Sun 2022). However, commonly used features in link prediction, such as CRISPR or local alignment scores, are either unavailable or too noisy for NCLDVs. Furthermore, the highly complex genome structures of eukaryotic hosts make it very difficult to extract informative features. Therefore, multi-class classification methods are preferred for NCLDV host prediction. These methods leverage heterogeneous, sequence-derived features to assign a host taxonomic label directly to a given virus, but face significant limitations when applied to large-scale NCLDV data. For instance, methods relying on protein language models, such as EvoMIL (Liu *et al.* 2024), are computationally prohibitive for large-scale metagenomics. Conversely, trait-only approaches such as RNAVirHost (Chen *et al.* 2024) are inadequate because relying solely on genomic traits overlooks the critical host-interaction signals encoded within viral proteins.

To fully exploit heterogeneous sequence-based features, we formulate NCLDV host prediction as a supervised learning problem, with carefully curated host groups serving as labels. Nevertheless, several challenges remain. First, highly confident host labels are largely restricted to a small number of cultivated reference genomes (e.g. those in RefSeq). Second, there is a profound discrepancy in data distribution between these well-annotated complete NCLDV genomes and the highly diverse, uncultivated Giant Virus Metagenome-Assembled Genomes (GVMAGs) recovered from natural environments. Consequently, standard supervised models trained solely on RefSeq data may fail to generalize to environmental contigs. Third, some existing tools output deterministic top-1 predictions. For fragmented metagenomic contigs, such overconfident point predictions can be highly misleading. Providing a statistically calibrated set of plausible hosts would be far more reliable and actionable for downstream ecological analyses.

**Figure 1:**
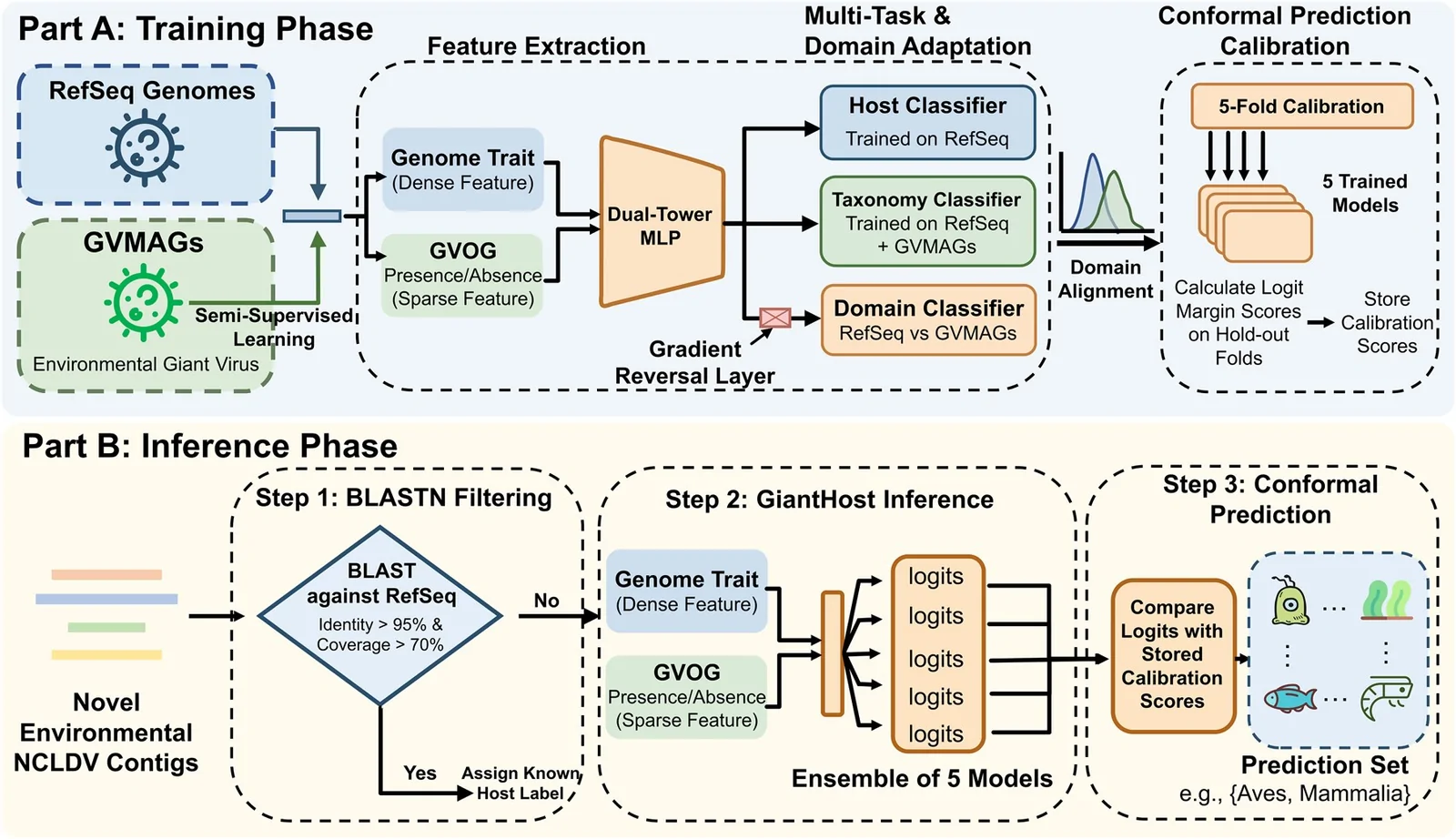
The overall architecture of GiantHost. (A) In the training phase, dense genomic traits and sparse GVOG features from RefSeq genomes and environmental GVMAGs are processed by a Dual-Tower MLP. A Gradient Reversal Layer enables domain adaptation between known and environmental viruses. A 5-fold calibration process computes margin scores for Conformal Prediction. Distinct from ensemble methods, this calibration step uses held-out data to quantify model uncertainty, enabling the model to construct a reliable set of candidate hosts with statistical guarantees. (B) During inference, novel contigs undergo a strict BLASTN pre-filtering. Unmatched sequences are processed by the 5-model. The Conformal Prediction module utilizes the stored calibration scores to output a multi-label Prediction Set, providing calibrated uncertainty quantification for host prediction.

To overcome these limitations, we propose GiantHost, a novel domain-adaptive and uncertainty-aware deep learning framework for hierarchical NCLDV host @prediction (Fig. 1). GiantHost is specifically designed to bridge the environmental gap and provide reliable annotations for metagenomic NCLDV contigs (>10 kb). Our contributions are threefold: (i) Dual-tower representation learning: we design a dual-tower Multilayer Perceptron (MLP) architecture to effectively fuse heterogeneous sequence features. One tower processes dense genomic traits, while the other handles high-dimensional, sparse functional profiles [over 8000-dimensional Giant Virus Orthologous Groups (Aylward *et al.* 2021) presence/absence vectors]. (ii) Semi-supervised multi-task learning with domain adaptation: to address data scarcity and domain shift, we leverage over 1400 high-quality environmental GVMAGs (Aylward *et al.* 2021). Although lacking host labels, these GVMAGs possess viral taxonomy annotations. We employ a multi-task learning strategy to co-predict host hierarchy and viral taxonomy. Crucially, we introduce a Domain Adversarial Neural Network (DANN) module (Ganin *et al.* 2016) with a gradient reversal layer to align the feature distributions of RefSeq genomes and environmental GVMAGs, effectively using GVMAGs as a bridge to improve generalization on environmental data. (iii) Conformal prediction for uncertainty quantification: instead of deterministic outputs, GiantHost wraps the hierarchical classification heads with Conformal Prediction (Angelopoulos and Bates 2023). By dynamically adjusting to the model’s uncertainty, it outputs prediction sets with a user-defined marginal recall guarantee (e.g. 90%), ensuring highly reliable host candidate screening. Furthermore, through model interpretability analysis via Integrated Gradients (Sundararajan *et al.* 2017) and extensive case studies on the North Atlantic Tara Oceans (Sunagawa *et al.* 2015) and Yangtze River metagenomes (Ren *et al.* 2025), we demonstrate GiantHost’s capacity to uncover biologically meaningful virus-host dynamics across diverse ecosystems.

## 2 Materials and methods

### 2.1 Dataset and feature representation

Formally, we frame the NCLDV host prediction task as a multi-class classification problem. Given a viral contig, our goal is to learn a mapping function $f:X_{\mathrm{dense}}\times X_{\mathrm{sparse}}\to Y$, where $X_{\mathrm{dense}}$ and $X_{\mathrm{sparse}}$ represent distinct feature modalities extracted from the sequence, and Y represents a discrete set of host categories.

To construct a high-quality and ecologically valid dataset for learning this mapping, we retrieved NCLDV genomes and their hosts from VirusHostDB (Mihara *et al.* 2016) and NCBI RefSeq (O’Leary *et al.* 2016). We strictly filtered out records based solely on *in vitro* permissive cell lines, retaining only hosts confirmed via natural infection or *in vivo* evidence. To prevent data leakage and redundancy, genomes were clustered at a 95% Average Nucleotide Identity (ANI) threshold using Vclust (Zielezinski *et al.* 2025), resulting in a curated dataset of 148 representative NCLDV genomes. They were fragmented into diverse contig lengths (5k, 10k, 15k, 20k bp), resulting in a final training corpus of 22 348 contigs.

**Figure 2:**
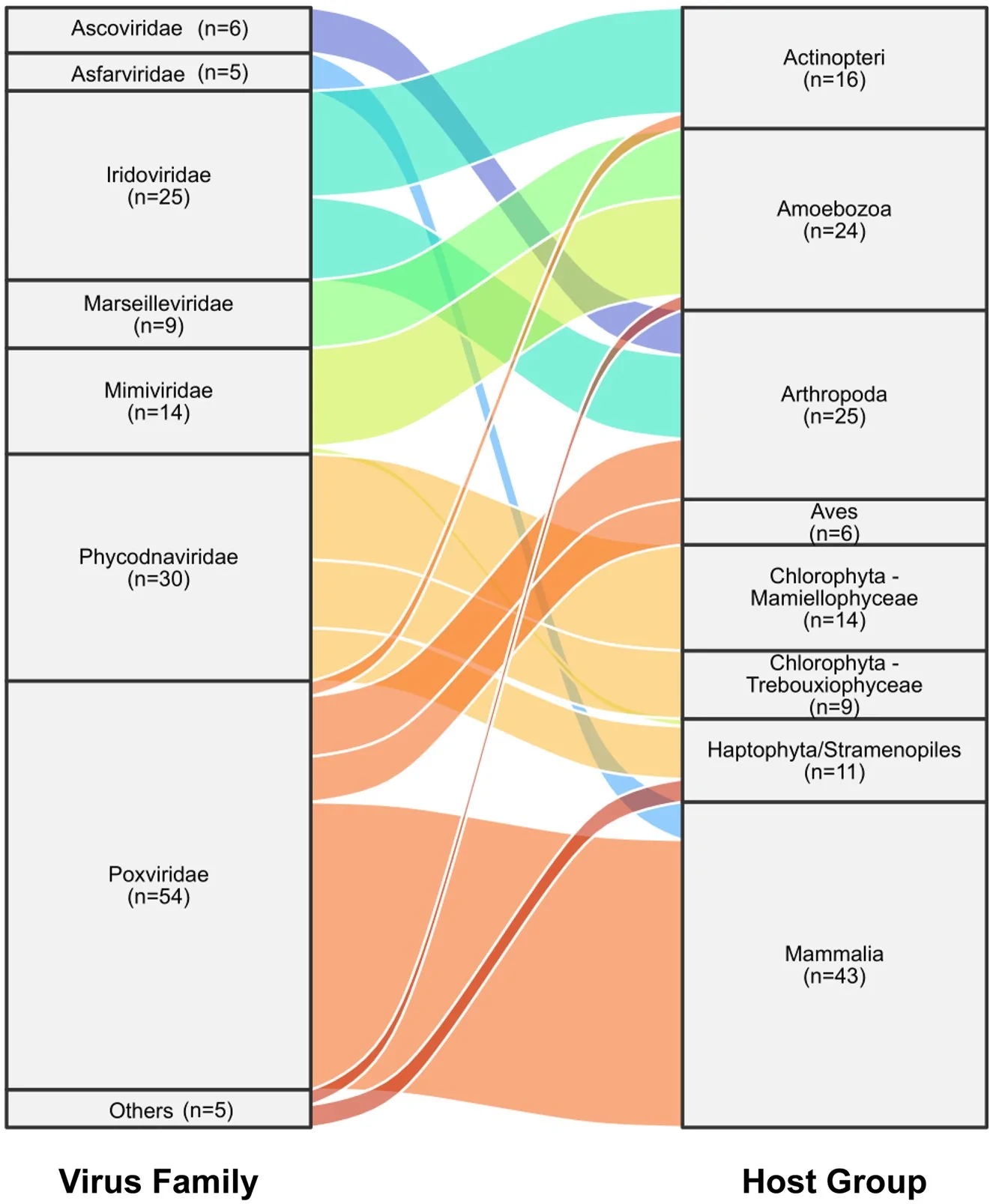
Distribution of associations between virus families and host groups across 148 records. To maintain visual clarity, categories with a total frequency of less than 3 are grouped as “Others”.

The curated dataset consists of 148 viral genomes distributed across 11 viral families. However, the raw host annotations for these viruses are highly granular and severely imbalanced, with many specific host taxa represented by only a few viral instances. To strike a balance between robust predictive coverage and biologically meaningful taxonomic resolution, we consolidated the original host taxonomy into eight ecologically and evolutionarily cohesive groups. Their associations with the viral families are shown in Fig. 2. While this consolidation captures distinct ecological niches and reduces extreme label sparsity, inherent biological representation biases persist: Mammalia constitutes nearly 30% of the dataset, whereas Aves accounts for only 4%. Detailed procedures for dataset curation, including the refinement of raw host annotations and genome fragmentation strategies, are provided in Supplementary Section S1.

We extracted two complementary feature modalities per viral contig to capture both sequence composition and functional repertoires. The first is a 137-dimensional dense vector of genomic traits (e.g. nucleotide/amino acid frequencies, codon usage) reflecting co-evolutionary signatures. The second is a sparse binary profile of approximately 8000 Giant Virus Orthologous Groups (GVOGs) representing specific phylogenetic landscapes and host-virus interactions. As an additional benchmark feature, tetranucleotide (*k* = 4) frequency vectors (256-d) are also evaluated, concatenated with trait in the continuous branch. Detailed extraction procedures are available in Supplementary Section S2.

### 2.2 The GiantHost architecture

GiantHost is specifically designed to integrate heterogeneous viral features while explicitly addressing the two most critical challenges in metagenomic viral host prediction: (i) the severe evolutionary and environmental distribution shift between well-characterized reference databases and uncultured environmental data, and (ii) the high risk of overconfident false-positive assignments when analyzing highly divergent, novel viruses. To address these, the GiantHost architecture comprises three synergistic modules: a hierarchical feature extractor, a domain adaptation network, and an uncertainty quantification layer, as shown in Fig. 1.

#### 2.2.1 Domain-adaptive multi-task learning framework

To process the heterogeneous dense genomic traits and sparse GVOG profiles, GiantHost employs a Dual-MLP architecture. This separate processing prevents sparse signals from being overshadowed by dense continuous variables, ultimately fusing them into a central, shared latent representation, z. This embedding z serves as the foundational hub for the entire architecture, upon which two simultaneous optimization strategies are applied. First, to ensure z captures biologically meaningful signals, we formulate a comprehensive multi-task learning framework. Distinct prediction heads branch out from z to simultaneously perform viral taxonomy classification and hierarchical host prediction (at both coarse-grained, e.g. metazoa vs. protist, and fine-grained levels). This joint optimization provides explicit multi-level supervision, anchoring z to both viral evolutionary lineages and concrete host associations. Second, to ensure z generalizes to unseen data, we address the domain shift between cultivated RefSeq genomes (source) and unannotated environmental GVMAGs (target) by passing z through a Domain-Adversarial Neural Network (DANN) module. By penalizing the model’s ability to distinguish between RefSeq and GVMAG origins (details of mechanical gradient reversal in Supplementary Section S3), the feature extractor is compelled to capture universal biological signatures. Consequently, the joint forces of multi-task supervision and adversarial training guarantee that the final representation z is highly discriminative for classification tasks yet strictly invariant to domain origin, effectively preventing out-of-distribution collapse on environmental data.

#### 2.2.2 Uncertainty quantification via conformal prediction

To rigorously quantify the uncertainty, GiantHost shifts from traditional single-label prediction to Conformal Prediction (CP), which leverages a held-out calibration set to empirically estimate the model’s error distribution, thereby allowing us to output a prediction set of plausible hosts with a mathematically guaranteed confidence level. Specifically, we define a nonconformity score s(x,y) that measures how much the model’s output disagrees with a candidate host *y*. Using the pre-softmax logits $\hat{z}$, we calculate the logit margin: $s(x,y)=\max_{y^{\prime }\neq y}\;{\hat{z}}_{y^{\prime }}-{\hat{z}}_{y}$ Intuitively, this score quantifies how much the network prefers other host candidates over *y*. By evaluating s(x,y) for the true labels within our calibration set, we construct an empirical distribution of nonconformity scores that reflects the model’s actual behavior on unseen data.

During inference for a novel viral contig $x_{\text{new}}$, instead of blindly trusting the top prediction, we evaluate every possible host *y*. We include *y* in our final prediction set $\Gamma^{\alpha }(x_{\text{new}})$ only if its nonconformity score falls within the (1−α) quantile (denoted as ${\hat{q}}_{\alpha }$) of our calibration distribution: $\Gamma^{\alpha }(x_{\text{new}})=\{y|s(x_{\text{new}},y)\leq {\hat{q}}_{\alpha }\}$ where α is the user-defined error rate, which is 0.1 by default. For well-characterized viruses, $\Gamma^{\alpha }$ seamlessly shrinks to a single, highly confident host. However, for highly divergent contigs, the set naturally expands to suggest multiple plausible candidates, or returns an empty set. This mechanism explicitly prevents the model from making confident but erroneous guesses on out-of-distribution data (detailed CP algorithms are provided in Supplementary Section S4).

### 2.3 Taxonomy-aware evaluation strategy and leakage prevention

Due to the extreme scarcity of gold-standard NCLDV-host associations, isolating a large, dedicated hold-out test set is impractical. Therefore, we rely on rigorous cross-validation frameworks. While we conducted standard 5-fold cross-validation, random splits inevitably suffer from homologous data leakage in genomic datasets. To rigorously simulate the real-world discovery of novel viruses, our primary evaluation emphasizes a taxonomy-aware Leave-One-Taxon-Out (LOTO) strategy. By strictly isolating all contigs of a held-out species (Leave-One-Species-Out, LOSO) or genus (Leave-One-Genus-Out, LOGO) into the test phase, we largely eliminate homology leakage and assess the model’s true generalization capability on highly divergent viruses. Furthermore, to prevent evaluation bias skewed by varying viral genome sizes, we introduce a genome-resolved accuracy metric. Specifically, each heldout genome is fragmented into contigs, and the model’s predictions are aggregated to compute an accuracy score for that individual genome. The final performance is reported as the macro-average of these per-genome accuracies (detailed formulation in Supplementary Section S6).

## 3 Results

We evaluated our framework through a comprehensive experimental pipeline. First, we benchmarked various model architectures and feature combinations with the BLAST filtering step completely excluded. Next, we validated the domain adaptation efficacy via Uniform Manifold Approximation and Projection (UMAP) and assessed the Conformal Prediction robustness by analyzing empirical recall and average set sizes across varying α thresholds. We further interpreted the biological relevance via protein-level feature attribution. Finally, we demonstrated the framework’s real-world ecological utility through case studies on the Tara Ocean and Yangtze River metagenomes.

### 3.1 Feature selection and model performance

To determine the optimal configuration for GiantHost, we systematically evaluated five machine learning architectures across seven combinations of genomic features. MLP and GNN take concatenated features directly as input. DualMLP and DualGNN use two MLP branches to separately encode discrete (GVOG) and continuous (kmer, trait, or combined) features; in DualGNN, the compressed branch outputs are concatenated as node embeddings for subsequent graph message passing. To rigorously assess the model’s generalization ability across different levels of taxonomic novelty, we conducted leave-one-species-out and leave-one-genus-out (Tables S1 and S2) cross-validations. Additionally, standard 5-fold cross-validation was performed, which demonstrated consistent performance trends (Table S3).

**Figure 3:**
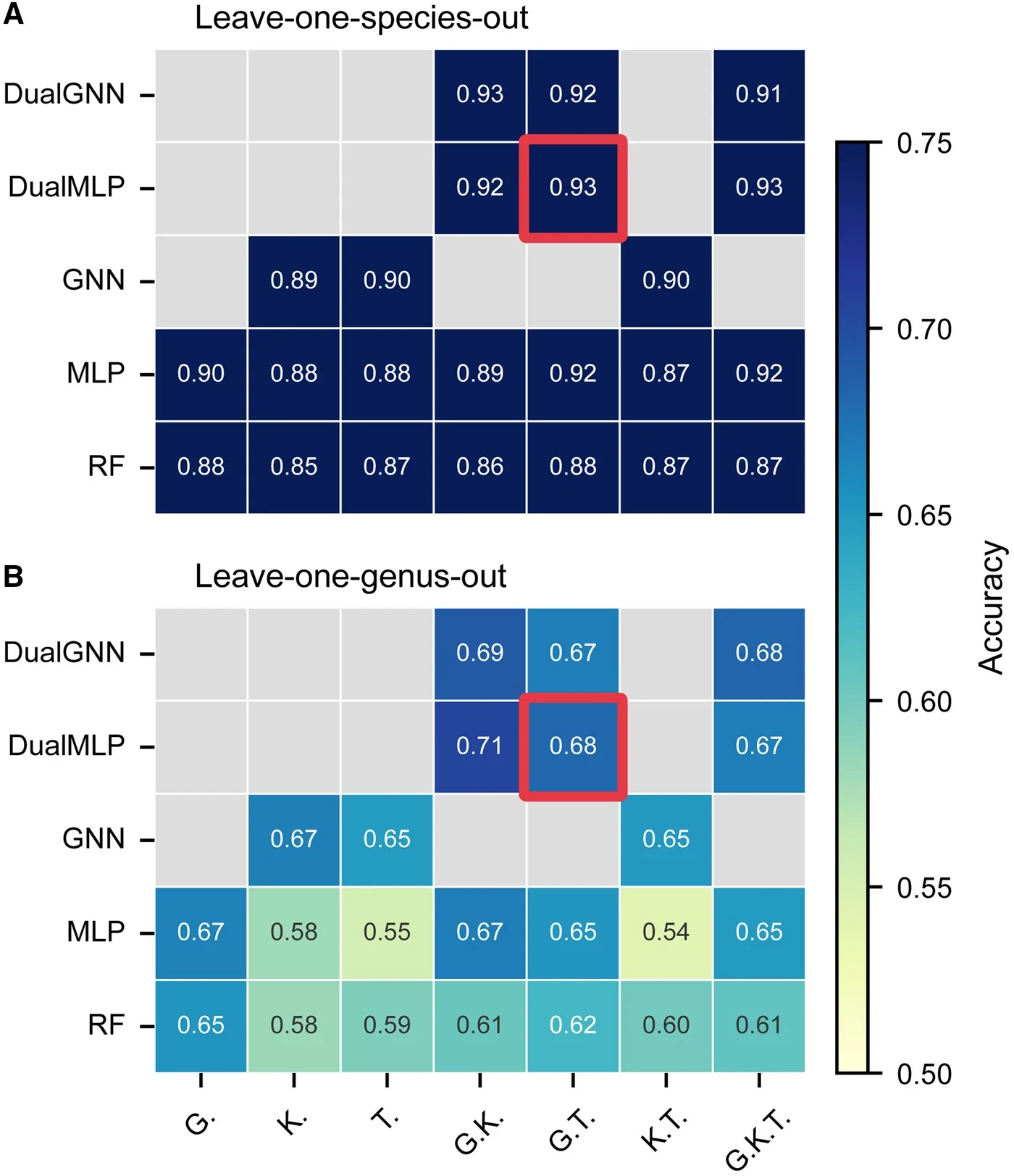
Host prediction performance of different models and feature combinations on 10 kbp NCLDV contigs across two evaluation strategies. (A) Host prediction accuracy evaluated using the leave-one-species-out setting. (B) Host prediction accuracy evaluated using the more stringent leave-one-genus-out setting. The heatmaps display the performance of five machine learning architectures (RF: Random Forest, MLP: Multilayer Perceptron, GNN: Graph Neural Network, DualMLP: Dual-tower MLP, DualGNN: Dual-tower GNN) across seven combinations of genomic features (G: GVOG, K: K-mer, T: Genome Trait). Here 4-mer is used. Grey cells represent untested configurations caused by dual-tower architectural constraints or intractable spatial complexity for GNNs using GVOGs.

Results on 10k bp contigs are shown in Fig. 3A and B. Deep learning models mostly outperformed the Random Forest baseline. Under the single-task setting, dual-tower architectures using the combination of GVOGs and K-mers (G.K.) achieved the highest accuracy. Regarding the model architecture, although DualGNN demonstrated competitive performance, the graph construction process introduces prohibitive memory and time complexities for large-scale metagenomic applications. Therefore, DualMLP was selected as the backbone. Compared to a standard single-tower MLP, the DualMLP naturally disentangles the high-dimensional, sparse presence/absence signals (GVOGs) from dense, continuous representations. This decoupling enhances the model’s robustness against incomplete contigs and provides a cleaner computational graph for downstream interpretability analysis (see Section 3.4). Crucially, the multi-task framework exposes the true generalization limits of different features by preventing overfitting to RefSeq’s cultivated viruses. Under this domain-shift stress test, the G.T. (GVOG + Trait) model exhibits exceptional stability, whereas the k-mer-based G.K. model suffers noticeable degradation (Fig. S2). This insight demonstrates that genomic traits are inherently more robust to environmental variations than sequence-level k-mers. Prioritizing real-world reliability, we established the multi-task DualMLP with G.T. as GiantHost’s default configuration.

### 3.2 Bridging the gap: domain adaptation

**Figure 4:**
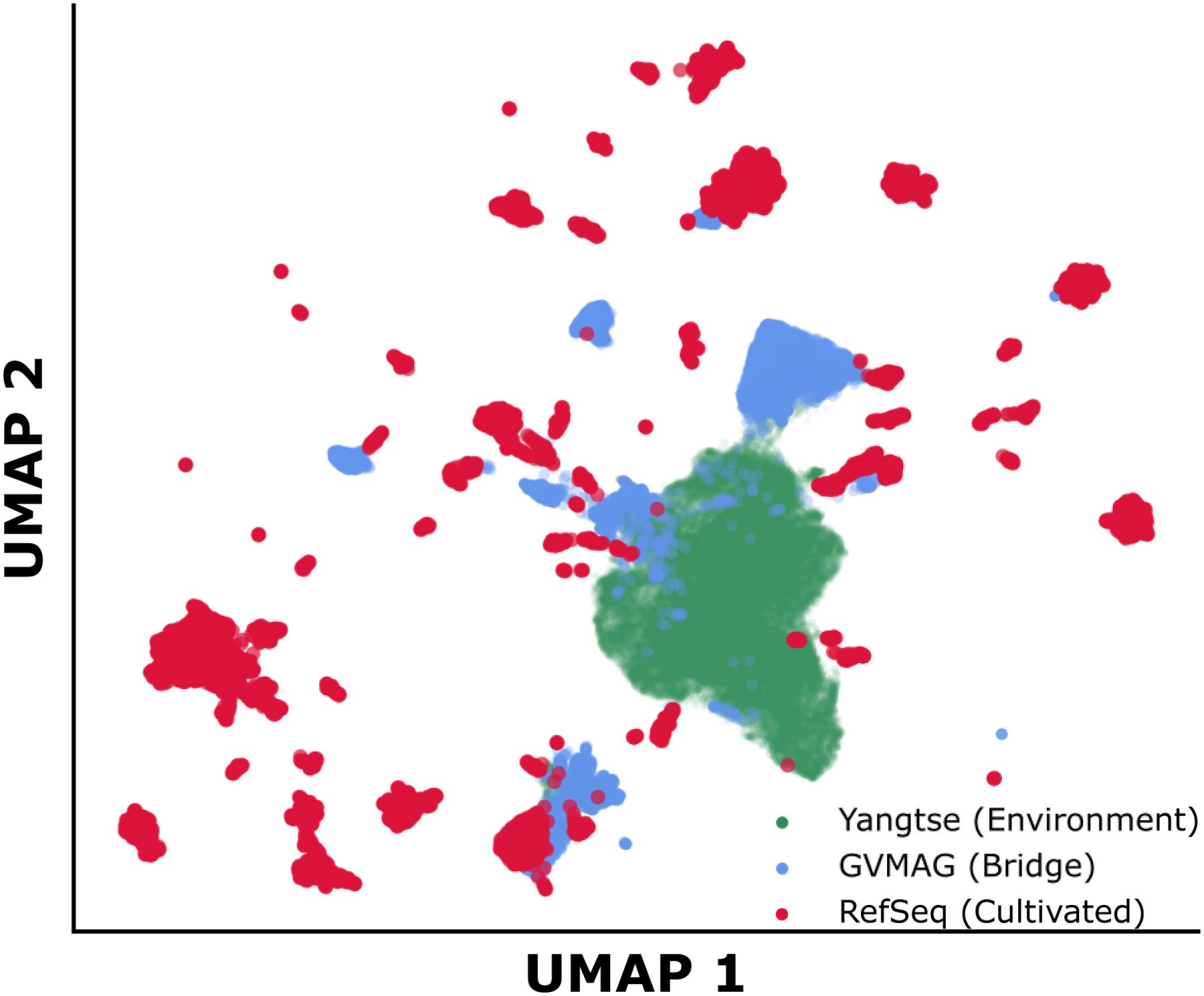
The scatter plot illustrates the feature representations of NCLDV contigs extracted from the penultimate layer of the GiantHost model. Red points represent cultivated RefSeq genomes, green points denote environmental contigs from the Yangtse River metagenome, and blue points indicate GVMAGs. The GVMAGs effectively act as an intermediate bridge, connecting the disparate feature spaces of the laboratory-isolated RefSeq dataset and the wild environmental dataset.

When predicting hosts for environmental NCLDVs, a major challenge is the severe distribution shift from RefSeq training data. To evaluate GiantHost’s ability to overcome this gap, we extracted the latent embeddings from the layer preceding the classification head and visualized them using UMAP (Fig. 4). The visualization reveals a stark separation between the RefSeq dataset (red), which is scattered into multiple distinct clusters representing diverse known host groups, and the Yangtse River environmental dataset (green), which forms a dense cluster with only partial overlap with a few RefSeq points. Remarkably, the semi-supervised GVMAGs (blue) successfully occupy the intermediate latent space, acting as a crucial topological bridge between the isolated cultivated viruses and the unexplored environmental viruses. The integration of a Domain-Adversarial Neural Network (DANN) module plays an essential role in forming this bridge. As demonstrated in our ablation study (Fig. S4), without adversarial training, the latent representations of GVMAGs tend to suffer from severe mode collapse, aggregating into an uninformative mass that fails to capture biological diversity. DANN mitigates this collapse by aligning feature distributions across domains.

### 3.3 Uncertainty-aware prediction

**Figure 5:**
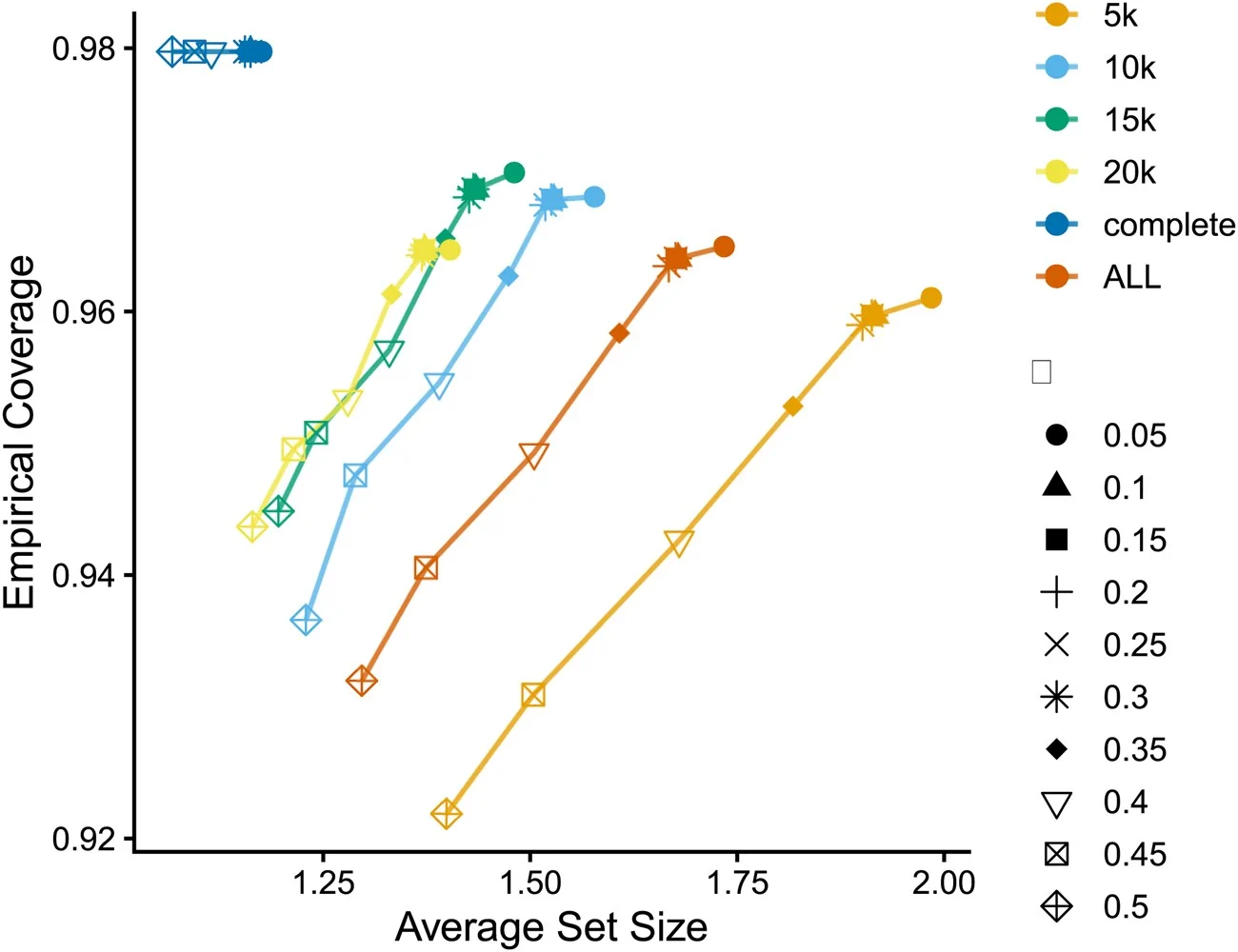
Performance trade-offs in Conformal Prediction. The plot illustrates the relationship between empirical coverage (defined as the proportion of contigs whose true hosts successfully captured by the prediction sets) and average set size across varying significance levels and contig lengths. Complete genomes achieve near-perfect coverage with minimal set sizes, while shorter contigs dynamically expand set sizes to maintain reliability.

To address model overconfidence on fragmented environmental contigs, GiantHost employs Conformal Prediction (CP) to output statistically rigorous prediction sets with a guaranteed marginal coverage of 1−α. We rigorously evaluated this using a Leave-One-Species-Out (LOSO) strategy coupled with 5-fold Cross-Conformal Prediction (CCP). Specifically, for each left-out species, the remaining data was partitioned into five folds to train five independent models, whose non-conformity scores were subsequently aggregated to construct the final prediction sets, thereby maximizing data utilization for both training and calibration. As depicted in the operating characteristic curves (Fig. 5), GiantHost exhibits a clear trade-off between empirical coverage and average set size across various sequence lengths and α thresholds. The model consistently meets or exceeds theoretical coverage requirements. Notably, the prediction set size dynamically adapts to the sequence information content: complete genomes yield highly confident, near-single-class predictions, whereas shorter fragments (e.g. 5k) adaptively expand the set size to maintain reliability. Furthermore, an in-depth analysis of predictions with a set size of two reveals that the co-predicted hosts are predominantly phylogenetically related or occupy overlapping ecological niches (e.g. Algae and Amoeba, or Mammalia and Aves). This demonstrates that the model captures meaningful biological ambiguities rather than making random errors (see Fig. S3). By outputting a statistically guaranteed shortlist rather than forcing a potentially erroneous single prediction, GiantHost effectively manages inherent uncertainty and significantly narrows the search space for downstream biological validation.

### 3.4 Biological interpretability

Using the Captum interpretability framework with Integrated Gradients (Sundararajan *et al.* 2017), we calculated feature attribution scores to identify the biological determinants driving GiantHost’s predictions (Fig. 7). For mammalian hosts, top predictors included immune evasion and membrane fusion proteins. Notably, GVOGm1255 (an interleukin receptor-like “decoy”) and poxvirus-specific fusion proteins (e.g. A33R, B22R) received high weights, reflecting viral strategies to neutralize cytokines and facilitate entry. Conversely, invertebrate (Arthropoda) predictions relied heavily on zinc-finger proteins (e.g. GVOG03078) critical for transcriptional hijacking. In marine ecosystems, viruses infecting *Mamiellophyceae* were strongly predicted by Auxiliary Metabolic Genes (AMGs) like thioredoxin/glutaredoxin (GVOG11645), highlighting the ecological necessity of viral-mediated redox regulation against photo-oxidative stress. For rigid-walled *Trebouxiophyceae*, the model prioritized enzymes vital for physical breaching (e.g. metallopeptidases, GVOG03080). Intriguingly, high scores were also assigned to uncharacterized proteins (e.g. GVOGm0199/DUF1390). This demonstrates GiantHost’s dual utility: validating established biological interactions while pinpointing novel, high-confidence lineage-specific viral proteins for future functional discovery.

### 3.5 Case study: Tara ocean NCLDV host landscape

To demonstrate the practical utility of GiantHost, we analyzed viral-enriched (0.22–1.6 µm) metagenomes from the Tara Oceans North Atlantic expedition (see Supplementary Section S10 for standard processing details and an additional Yangtze River case study). Putative NCLDV contigs were identified by GiantHunter (Qu *et al.* 2025) and subsequently fed into GiantHost for host prediction. Crucially, leveraging our conformal prediction framework, contigs yielding empty or highly uncertain (multiple) prediction sets were strictly classified as “Unknown” from the outset to minimize false positives.

**Figure 6:**
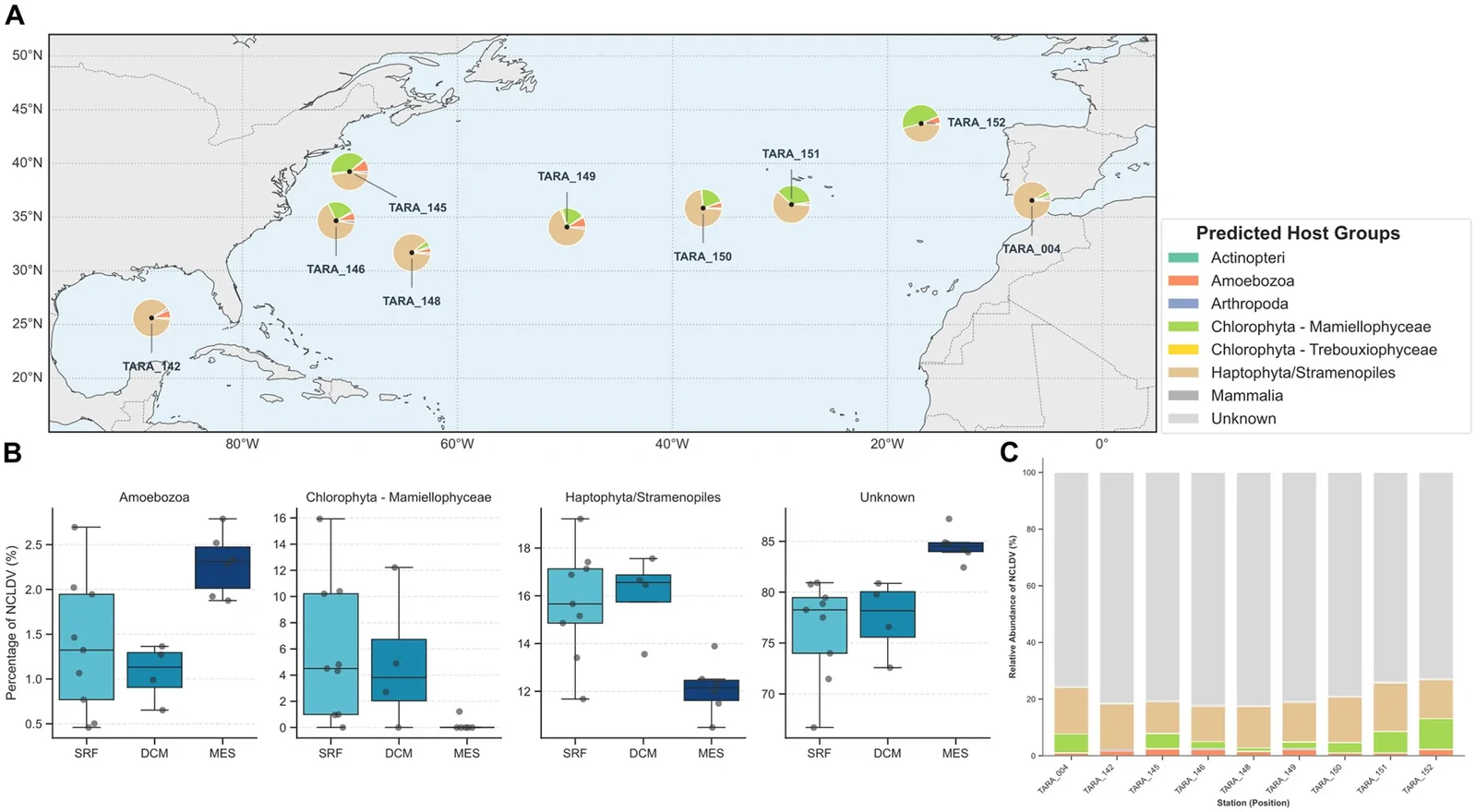
Biogeographical and vertical distribution of NCLDV hosts in the North Atlantic Ocean predicted by GiantHost. (A) Map of the North Atlantic showing the relative abundance of predicted NCLDV host groups at the surface of various Tara Oceans stations. (B) Vertical stratification of NCLDV relative abundance across different oceanic depth layers: Surface (SRF), Deep Chlorophyll Maximum (DCM), and Mesopelagic (MES). Boxplots show the distribution of specific host groups (Amoebozoa, Chlorophyta—Mamiellophyceae, Haptophyta/Stramenopiles) and the “Unknown” category (predictions rejected by the conformal prediction framework due to high uncertainty). (C) Stacked bar charts illustrating the relative abundance composition of predicted NCLDV host groups across the sampled stations.

Applying GiantHost across oceanic depth profiles revealed a distinct vertical stratification of predicted hosts (Fig. 6B). Viruses predicted to infect photosynthetic algae (*Chlorophyta—*Mamiellophyceae and *Haptophyta*/Stramenopiles) exhibit high relative abundance in the sunlit Surface (SRF) and Deep Chlorophyll Maximum (DCM) layers, followed by a drastic decline in the aphotic Mesopelagic (MES) zone. Conversely, viruses infecting *Amoebozoa*—typically heterotrophic protists—show a substantially relative increase in the MES layer. While viruses associated with photosynthetic hosts still maintain a higher overall proportion than those of *Amoebozoa* in the deep ocean, this relative enrichment reflects the growing ecological importance of heterotrophic interactions in the aphotic microbial food web. Notably, the proportion of these “Unknown” NCLDVs notably increases in the MES layer (Fig. 6B, rightmost panel). This observation not only reflects the vast, unexplored viral “dark matter” prevalent in the deep sea (Moniruzzaman *et al.* 2020, Schulz *et al.* 2020), but also highlights the robustness of our uncertainty-aware approach: rather than forcing erroneous top-1 predictions for highly divergent deep-sea viruses, GiantHost correctly flags them, thereby reliably guiding future targeted discoveries.

**Figure 7:**
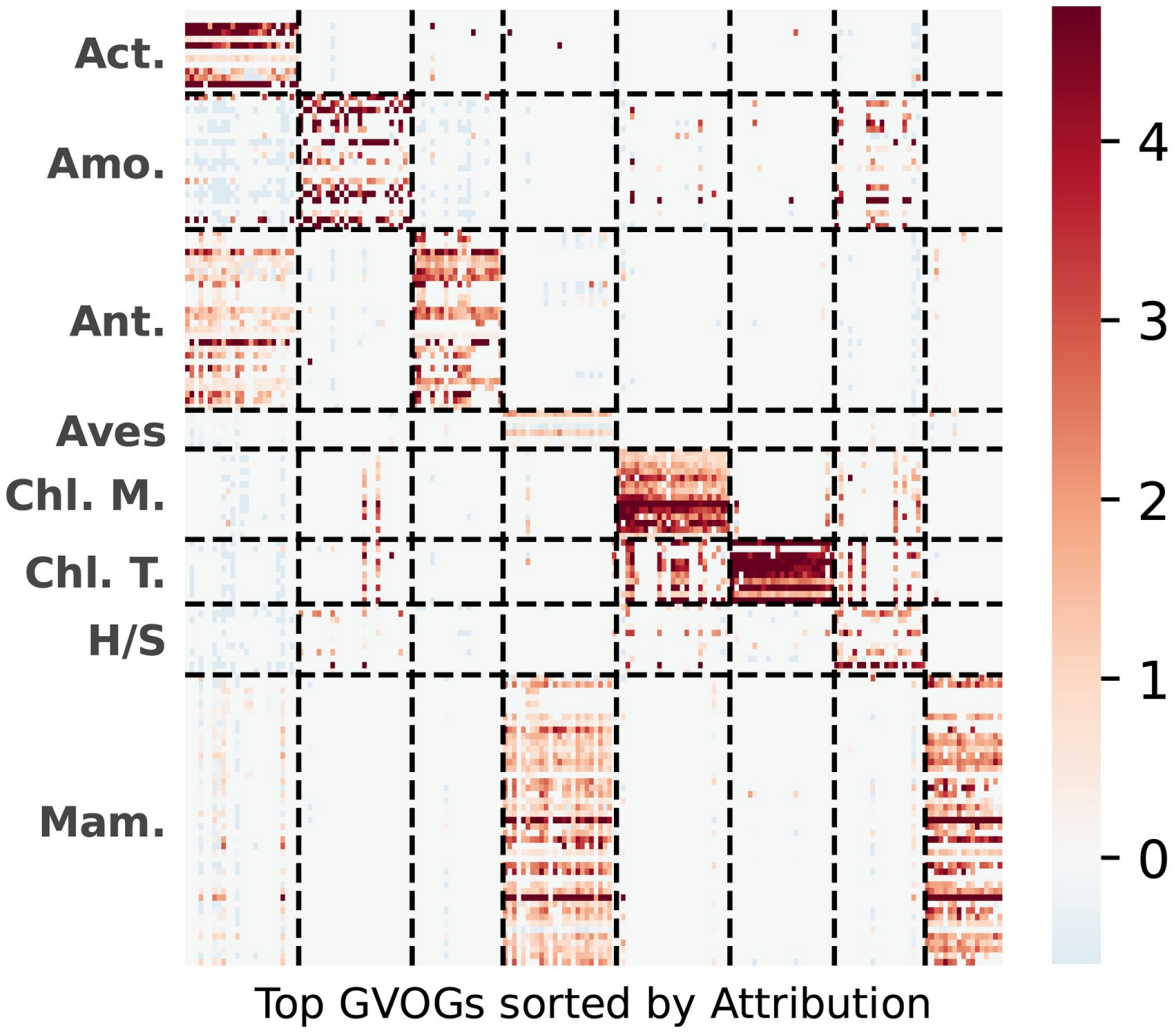
Feature attribution scores of the top 25 distinguishing GVOGs across 8 host lineages: Actinopteri(Act.), Amoebozoa(Amo.), Arthropoda(Art.), Aves, Chlorophyta-Mamiellophyceae(Chl.M.), Chlorophyta-Trebouxiophyceae(Chl.T.), Haptophyta/Stramenopiles(H./S.), Mammalia(Mam.). Darker colors indicate stronger positive contributions. The model captures lineage-specific viral arsenals, from mammalian immune evasion to algal auxiliary metabolic genes.

## 4 Discussion

In this study, we present GiantHost, a deep learning framework for predicting NCLDV host. To overcome the limitations of traditional alignment-based methods and the severe domain shift between cultivated and environmental viruses, GiantHost integrates a Dual-MLP architecture with domain-adversarial and multi-task learning. This design captures critical protein-level signals while ensuring robust generalization. Furthermore, its application to global ocean datasets successfully recovered biologically meaningful ecological dynamics. Finally, by incorporating Conformal Prediction, GiantHost mitigates model overconfidence—explicitly flagging highly divergent contigs as “Unknown” to provide a conservative, uncertainty-aware baseline for future discoveries.

Despite its robust performance, GiantHost exhibits partial confusion between certain host clades, notably misclassifying some Haptophyta/Stramenopiles-infecting contigs as Amoebozoa-infecting. This partly reflects a limitation of our sequence-based approach in resolving host clades with overlapping genomic signatures. This challenge may be compounded by the chimeric nature of giant virus genomes: amoebae act as intracellular “melting pots” where diverse microorganisms co-exist and exchange genes (Boyer *et al.* 2009), potentially generating shared genomic features between Amoebozoa-infecting and algal-infecting NCLDV lineages. Future work will transition to a virus–host bipartite graph link-prediction framework integrating both NCLDV and host genomic features, and will scale as more gold-standard pairs become available.

## Author contributions

Fuchuan Qu (Conceptualization, Data curation, Formal analysis, Investigation, Methodology, Software, Visualization, Writing—original draft, Writing—review & editing), Guowei Chen (Conceptualization, Investigation, Writing—review & editing), and Yanni Sun (Project administration, Supervision, Writing—review & editing)

## Supplementary Material

btag498_Supplementary_Data

## Data Availability

Data is available at https://github.com/FuchuanQu/GiantHost.
