# Supplementary material for: GiantHost: a domain-adaptive and uncertainty-aware framework for giant virus host prediction
Source: Bioinformatics. 2026 Aug 21;42(Suppl 2):btag498. doi: 10.1093/bioinformatics/btag498 (PMC13501317; doi:10.1093/bioinformatics/btag498)
Supplement: Supplementary file 1 [file btag498_supplementary_data.pdf]

GiantHost: Supplementary Documents

1. DETAILED PROCEDURES FOR DATA CLEANING AND TAXONOMIC LABEL MERGING

To construct a high-quality dataset for GiantHost, we initially retrieved viral records from the VirusHostDB. Using "Nucleocytoviricota" as the keyword for the virus lineage, we identified 189 viral records. After excluding 5 records lacking explicit host information, 184 unique viruses were retained. Due to the multi-host nature of certain viruses, these corresponded to 331 virus-host interaction pairs. To ensure standardized taxonomic classification, we utilized the NCBITaxa module from the ete3 Python library to systematically annotate and map the full taxonomic lineages for both viruses and their corresponding hosts based on their NCBI Taxonomy IDs.

Viral genomes were downloaded using their corresponding RefSeq IDs. For viruses with multiple associated genomic sequences or fragmented contigs, a rigorous manual screening and quality control pipeline was implemented. We utilized CheckV to assess genome completeness. Sequences flagged as "UNVERIFIED" or those representing only single genes rather than complete or near-complete genomes were strictly discarded to prevent the introduction of incomplete genomic features into the model training phase.

To avoid data leakage and model bias caused by highly redundant sequences, while simultaneously preserving valuable genetic diversity within the limited dataset of giant viruses, we applied a sequence similarity-based deduplication strategy using vclust. The rules were defined as follows:

- High Similarity (>95%): For genome pairs exhibiting greater than 95% similarity (e.g., GU244497 and NC_014637; NC_044943 and NC_044957), only one representative sequence was retained. Priority was given to sequences with the "NC_" prefix (indicating curated RefSeq status) and longer sequence lengths.
- Moderate Similarity (<95%): Given the relative scarcity of complete Nucleocytoviricota genomes, sequences with less than 95% similarity were both retained to capture genetic variations.
- Sub-sequence Resolution: Cases where one sequence was a direct substring of another (e.g., NC_043228) were resolved by retaining only the complete parent sequence.

A critical challenge in virus-host data curation is distinguishing true ecological hosts from artificial laboratory hosts. During our manual curation, we encountered instances where a single virus was annotated with multiple hosts from distinct taxonomic groups (e.g., Ambystoma and Cyprinus carpio for ATV). Upon detailed literature review, we identified that while the virus naturally infects Ambystoma (salamander), the infection of Cyprinus carpio (carp) was an artifact of using the Epithelioma papulosum cyprini (EPC) permissive cell line in vitro. The genomic signatures of viruses are profoundly shaped by long-term co-evolution with their natural hosts in vivo, involving complex immune evasion and physical barrier penetration. While permissive cell lines (e.g., EPC or HeLa) lack these systemic biological barriers and allow broad in vitro infections, they do not reflect true ecological virus-host interactions. Therefore, to ensure the ecological validity of GiantHost, we established a strict filtering principle: only natural infections or in vivo experimental infections supported by primary literature were retained. All in vitro cell culture records were systematically excluded from the host label set. For arthropod-borne viruses, databases sometimes list the vector as a host. For instance, in the case of the African Swine Fever Virus (ASFV), records of the tick vector (Ornithodoros) were manually removed. We exclusively retained the mammalian host records (e.g., Suidae), as the primary disease pathology and long-term genomic adaptation of the virus are primarily driven by its mammalian hosts rather than its transient vectors.

To ensure robust model training while preserving meaningful biological resolution, we meticulously curated and merged the host taxonomic labels based on both data availability and evolutionary ecology. For well-represented lineages, we maintained fine-grained taxonomic levels. Specifically, mammalian and ray-finned fish hosts were categorized at the class level

(Mammalia and Actinopteri, respectively). The Amoebozoa group was maintained at the phylum level, which in our dataset is predominantly represented by the genus *Acanthamoeba* (e.g., *Acanthamoeba polyphaga* and *Acanthamoeba castellanii*), serving as a critical representative of free-living amoeba hosts.

For the phylum Arthropoda, the dataset comprised 22 records of Insecta, 2 of Malacostraca, and 1 of Branchiopoda. Rather than discarding the three rare crustacean records to strictly form an Insecta group, we aggregated them at the phylum level (Arthropoda). This decision is biologically highly justified: Arthropoda is a monophyletic clade sharing deeply conserved physiological traits, such as chitinous exoskeletons, and homologous innate immune responses, including the highly conserved Toll and Imd pathways. Consequently, viruses infecting crustaceans and insects face similar intracellular environments and selective pressures, resulting in shared viral genomic signatures. Merging these records preserves valuable viral genomic diversity and allows GiantHost to learn generalized arthropod-infecting features rather than overfitting to insect-specific traits.

Conversely, we adopted a splitting strategy for the phylum Chlorophyta, separating it into two distinct class-level groups: Mamiellophyceae and Trebouxiophyceae. Preliminary experiments utilizing a baseline Random Forest model demonstrated that the viral genomes infecting these two classes possess highly distinct features and can be easily distinguished. To maximize the taxonomic resolution and predictive capability of our tool, we maintained them as separate host groups, reflecting their divergent virus-host co-evolutionary trajectories.

Finally, we encountered significant feature overlap between the phylum Haptophyta and various classes within the Stramenopiles (including Bigyra, Pelagophyceae, Phaeophyceae, and Raphidophyceae). Preliminary models struggled to differentiate viruses infecting these two groups, with approximately 40% cross-misclassification. Rather than a computational limitation, this reflects a profound biological reality. Both Haptophyta and Stramenopiles are major eukaryotic phytoplankton groups that are evolutionarily linked (historically grouped within the Chromalveolata supergroup) and share complex plastids derived from the secondary endosymbiosis of red algae. Because they occupy highly similar marine ecological niches and possess fundamentally similar cellular metabolic networks, the large DNA viruses infecting them experience identical selective pressures. This drives convergent evolution in viral genomic signatures, such as codon usage and auxiliary metabolic genes. Therefore, forcing an artificial boundary between them would contradict ecological reality. We merged them into a single functional and ecological super-group (Haptophyta/Stramenopiles), which significantly enhanced model stability and accurately reflects the natural continuum of virus-host interactions in aquatic ecosystems.

2. MATHEMATICAL FORMULATION OF FEATURE EXTRACTION

To capture the multifaceted evolutionary signatures of *Nucleocytoviricota* genomes shaped by their hosts, GiantHost extracts three distinct categories of features from each viral genome: basic genomic traits, Giant Virus Orthologous Groups (GVOGs), and k-mer frequencies. Let a viral genome sequence be denoted as $S = s_1 s_2 \dots s_L$, where $s_i \in \Sigma = \{\text{A, C, G, T}\}$ and L is the total length of the genome. The final feature representation of the genome is a concatenated vector of these three components.

A. Comprehensive Genome Traits

Rather than relying solely on simple metrics like GC content, GiantHost constructs a meticulously engineered 137-dimensional genome trait vector, $\mathbf{f}_{\text{trait}} \in \mathbb{R}^{137}$. This vector serves as a comprehensive "compositional fingerprint" of the viral genome, capturing evolutionary signatures at the DNA, RNA (codon), and protein levels. These compositional biases are profoundly shaped by the host's intracellular environment, mutational pressures, and translational machinery. The 137 features are mathematically formulated into four distinct sub-spaces:

Nucleotide Frequencies (4 dimensions): The foundational composition of the viral genome is represented by the global frequencies of the four standard nucleotides. Let $S = s_1 s_2 \dots s_L$ be the viral genome sequence of length L, where $s_i \in \Sigma_{\text{nu}} = \{\text{A, C, G, T}\}$. The frequency of each nucleotide $x \in \Sigma_{\text{nu}}$ is defined as:

$$f_{\text{nu}}(x) = \frac{1}{L} \sum_{i=1}^{L} \mathbb{I}(s_i = x)$$

Global and Context-Dependent Dinucleotide Biases (48 dimensions): Dinucleotide relative abundances are strong indicators of host-induced mutational pressures (e.g., CpG depletion due to host DNA methylation and subsequent deamination). We extract 16 global dinucleotide frequencies (f_{dinu}). Furthermore, to capture the intricate constraints of the host's translational machinery, we extract 32 context-dependent dinucleotide frequencies from the coding regions: bridging dinucleotides ($f_{\text{dinu\_bdg}}$, spanning the 3rd position of one codon and the 1st position of the next) and non-bridging dinucleotides ($f_{\text{dinu\_non\_bdg}}$, positions 1-2 and 2-3 within a codon). For any dinucleotide xy, the general frequency within a specific index set $\mathcal{I}$ is formulated as:

$$f_{\text{context\_dinu}}(xy) = \frac{1}{|\mathcal{I}|} \sum_{i \in \mathcal{I}} \mathbb{I}(s_i s_{i+1} = xy)$$

where $\mathcal{I}$ encompasses all valid indices for global, bridging, or non-bridging contexts, respectively.

Codon Usage Bias (64 dimensions): Viruses are obligate intracellular parasites that must hijack the host's tRNA pool for protein synthesis. Consequently, viral codon usage is under strong selective pressure to align with the host's codon optimality. Let $\mathcal{C}(S)$ be the set of all codons extracted from the predicted coding sequences of S, with total count N_c. For each of the 64 possible codons c, its usage bias is calculated as:

$$f_{\text{codon}}(c) = \frac{1}{N_c} \sum_{j=1}^{N_c} \mathbb{I}(\text{codon}_j = c)$$

4. Amino Acid Usage Bias (21 dimensions): Finally, the amino acid composition reflects the functional and structural adaptation of the viral proteome to the host's biochemical environment (e.g., pH, temperature, and metabolic availability). Let $\mathcal{A}(S)$ be the translated amino acid sequence comprising N_a residues. For each standard amino acid a (including the stop codon/gap denoted as a space), the frequency is:

$$f_{\text{aa}}(a) = \frac{1}{N_a} \sum_{j=1}^{N_a} \mathbb{I}(\text{aa}_j = a)$$

The final genome trait vector is the concatenation of these four compositional profiles:

$$\mathbf{f}_{\text{trait}} = \mathbf{f}_{\text{nu}} \oplus \mathbf{f}_{\text{dinu}} \oplus \mathbf{f}_{\text{dinu\_bdg}} \oplus \mathbf{f}_{\text{dinu\_non\_bdg}} \oplus \mathbf{f}_{\text{codon}} \oplus \mathbf{f}_{\text{aa}}$$

This 137-dimensional vector explicitly embeds the multi-layered virus-host co-evolutionary signals into the node features of the GiantHost model.

B. Giant Virus Orthologous Groups

To capture the functional and evolutionary repertoire of the viruses, we utilize giant Virus Orthologous Groups (GVOGs). Let $\mathcal{V} = \{V_1, V_2, \ldots, V_M\}$ represent the reference database consisting of M distinct orthologous groups. For a given viral genome S, let $\mathcal{P}(S)$ denote the set of all predicted protein-coding sequences (CDS) derived from S.

The GVOG feature is represented as a high-dimensional vector $\mathbf{f}_{\text{GVOG}} \in \mathbb{N}^M$, where each element corresponds to the presence or abundance of a specific orthologous group. The j-th component of this vector is defined as the count of proteins in S that are homologous to the orthologous group V_j:

$$\mathbf{f}_{\text{GVOG}}^{(j)} = \sum_{p \in \mathcal{P}(S)} \mathbb{I}(\text{map}(p, V_j))$$

where $\text{map}(p, V_j)$ is a boolean function that returns true if protein p is successfully annotated to the orthologous group V_j (e.g., via HMMER searches exceeding a predefined significance threshold). This gene-content profile effectively captures the auxiliary metabolic genes and core replication machinery adapted to specific host environments.

C. k-mer Frequencies

Oligonucleotide frequencies (k-mers) are powerful proxies for host-induced mutational pressures, codon usage biases, and restriction-modification system avoidance. For a given k-mer length k, there are $|\Sigma|^k = 4^k$ possible distinct k-mers. Let $\mathcal{W} = \{w_1, w_2, \ldots, w_{4^k}\}$ be the set of all possible k-mers.

For any specific k-mer $w_j \in \mathcal{W}$, its absolute count $C(w_j, S)$ within the genome S is calculated by sliding a window of size k across the sequence:

$$C(w_j, S) = \sum_{i=1}^{L-k+1} \mathbb{I}(s_i s_{i+1} \ldots s_{i+k-1} = w_j)$$

To account for varying genome lengths, the counts are normalized into relative frequencies. The k-mer feature vector $\mathbf{f}_{\text{kmer}} \in \mathbb{R}^{4^k}$ is constructed such that its j-th element is:

$$\mathbf{f}_{\text{kmer}}^{(j)} = \frac{C(w_j, S)}{L - k + 1}$$

We have conducted a sensitivity analysis on the choice of k value. Using the default MLP model, we tested the leave-one-species-out and leave-one-genus-out accuracies on different k values, as shown in Figure S1. Considering the tradeoff between computational cost and accuracy, we choose k=4.

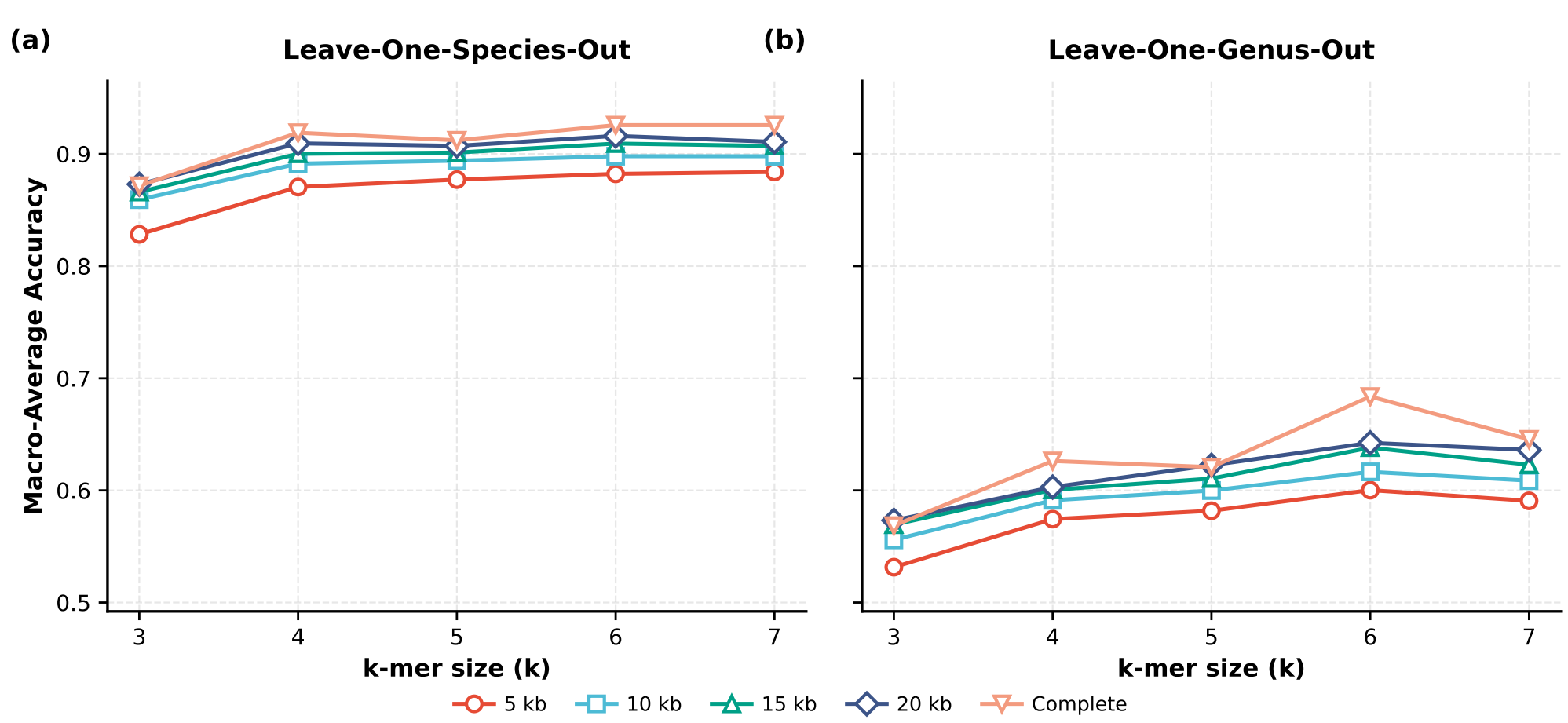

Fig. S1. Kmer sensitivity analysis on leave-one-species-out and leave-one-genus-out

D. Final Feature Integration

The comprehensive feature representation for the viral genome S, denoted as $\mathbf{X}_S$, is the concatenation ($\oplus$) of the three feature spaces:

$$\mathbf{X}_S = \mathbf{f}_{\text{trait}} \oplus \mathbf{f}_{\text{GVOG}} \oplus \mathbf{f}_{\text{kmer}}$$

This unified vector $\mathbf{X}_S \in \mathbb{R}^{d_1+M+4^k}$ serves as the input feature for the subsequent neural network.

3. DETAILED FORMULATION OF THE DOMAIN-ADVERSARIAL NEURAL NETWORK (DANN)

In this section, we provide the formal mathematical definition and optimization strategy for the Domain-Adversarial Neural Network (DANN) module integrated into GiantHost. The primary objective of this module is to mitigate the distribution shift between the cultivated viral genomes and the environmental Metagenome-Assembled Genomes (GVMAGs).

Let the source domain (RefSeq contigs with known labels) be denoted as $\mathcal{D}_S = \{(\mathbf{x}_i^s, y_i^s)\}_{i=1}^{N_s}$, where $\mathbf{x}_i^s$ represents the input features and y_i^s represents the corresponding biological labels (e.g., host taxonomy). Let the target domain (unannotated environmental GVMAGs) be denoted as $\mathcal{D}_T = \{\mathbf{x}_j^t\}_{j=1}^{N_t}$. For domain classification, we assign a domain label $d_i = 0$ for samples originating from $\mathcal{D}_S$ and $d_i = 1$ for samples from $\mathcal{D}_T$.

The architecture consists of three key components:

1. **Feature Extractor** $G_f(\cdot;\theta_f)$: Maps the heterogeneous input features to a shared latent representation $\mathbf{z} \in \mathbb{R}^d$, such that $\mathbf{z} = G_f(\mathbf{x};\theta_f)$.
2. **Label Predictor** $G_y(\cdot;\theta_y)$: Predicts the biological labels from the latent representation, yielding $\hat{y} = G_y(\mathbf{z};\theta_y)$.
3. **Domain Classifier** $G_d(\cdot;\theta_d)$: Predicts the domain origin from the latent representation, yielding $\hat{d} = G_d(\mathbf{z};\theta_d)$.

To achieve domain adaptation, we aim to learn a feature representation $\mathbf{z}$ that is discriminative for the main classification tasks but invariant with respect to the domain shift. This is formulated as a minimax optimization problem. The prediction loss $\mathcal{L}_y$ and the domain classification loss $\mathcal{L}_d$ are defined as follows:

$$\mathcal{L}_y(\theta_f, \theta_y) = \frac{1}{N_s} \sum_{i=1}^{N_s} L_y \left(G_y(G_f(\mathbf{x}_i^s;\theta_f);\theta_y), y_i^s \right)$$

$$\mathcal{L}_d(\theta_f, \theta_d) = \frac{1}{N_s + N_t} \sum_{i=1}^{N_s+N_t} L_d \left(G_d(G_f(\mathbf{x}_i;\theta_f);\theta_d), d_i \right)$$

where L_y is the cross-entropy loss for the multi-task hierarchical predictions, and L_d is the binary cross-entropy loss for domain discrimination. The overall objective function is:

$$\mathcal{E}(\theta_f, \theta_y, \theta_d) = \mathcal{L}_y(\theta_f, \theta_y) - \lambda \mathcal{L}_d(\theta_f, \theta_d)$$

where $\lambda > 0$ is the adaptation parameter that controls the trade-off between the two objectives. We seek the optimal parameters $\hat{\theta}_f, \hat{\theta}_y, \hat{\theta}_d$ that satisfy:

$$(\hat{\theta}_f, \hat{\theta}_y) = \arg\min_{\theta_f, \theta_y} \mathcal{E}(\theta_f, \theta_y, \hat{\theta}_d)$$

$$\hat{\theta}_d = \arg\max_{\theta_d} \mathcal{E}(\hat{\theta}_f, \hat{\theta}_y, \theta_d)$$

To optimize this minimax objective using standard gradient descent algorithms, we introduce a Gradient Reversal Layer (GRL). During the forward pass, the GRL acts as an identity transform. During the backpropagation pass, it multiplies the gradient by a negative constant $-\lambda$. Formally, the GRL function $\mathcal{R}(\mathbf{x})$ is defined by its forward and backward behaviors:

$$\mathcal{R}(\mathbf{z}) = \mathbf{z} \quad \text{(Forward pass)}$$

$$\frac{\partial \mathcal{R}}{\partial \mathbf{z}} = -\lambda \mathbf{I} \quad \text{(Backward pass)}$$

where $\mathbf{I}$ is the identity matrix. By inserting the GRL between the feature extractor G_f and the domain classifier G_d, the pseudo-function optimized by standard backpropagation becomes:

$$\tilde{\mathcal{E}}(\theta_f, \theta_y, \theta_d) = \mathcal{L}_y(\theta_f, \theta_y) + \mathcal{L}_d(\theta_f, \theta_d)$$

Consequently, the gradients updating the feature extractor parameters θ_f are computed as:

$$\frac{\partial \tilde{\mathcal{E}}}{\partial \theta_f} = \frac{\partial \mathcal{L}_y}{\partial \theta_f} - \lambda \frac{\partial \mathcal{L}_d}{\partial \theta_f}$$

This ensures that the feature representations are optimized to minimize the prediction error while simultaneously maximizing the domain classification error, thereby successfully aligning the feature distributions of the RefSeq and GVMAG domains.

4. THEORETICAL FRAMEWORK OF CONFORMAL PREDICTION IN GIANTHOST

To provide statistically rigorous uncertainty quantification for the hierarchical viral host prediction, we implemented a cross-fitted pooled Conformal Prediction (CP) framework. The taxonomy is structured into two levels: Level 1 ($\mathcal{Y}_1 = \{\text{Metazoa}, \text{Protist}\}$) and Level 2 ($\mathcal{Y}_2$, comprising 8 specific host groups). We define $\mathcal{C}(y_1)$ as the set of Level 2 classes belonging to the Level 1 branch y_1.

Instead of using the Adaptive Prediction Set (APS) heuristic, we employ the **Logit Margin** as the non-conformity measure for Level 2 predictions. For a given contig x and a candidate class $y \in \mathcal{C}(y_1)$, let z_c denote the logit output of the neural network for class c. The logit margin score is defined as:

$$s_{\text{margin}}(x, y) = \max_{c \in \mathcal{C}(y_1) \setminus \{y\}} z_c - z_y$$

A higher margin score indicates that the model assigns a higher logit to a competing class than to the candidate class y, implying lower conformity. For Level 1, we utilize the inverse probability score $s_1(x, y_1) = 1 - \sum_{c \in \mathcal{C}(y_1)} \hat{p}_c(x)$, where $\hat{p}$ represents the softmax probabilities.

The detailed procedures for cross-fitted calibration and hierarchical set prediction are outlined in **Algorithm 1** and **Algorithm 2**.

Algorithm S1. Cross-Fitted Hierarchical Calibration with Logit Margin

---

Require: Training genomes $\mathcal{D}_{\text{train}}$, number of folds K, window size L, stride S.
Ensure: Trained models $\{f_k\}_{k=1}^{K}$, calibration score banks $\mathcal{S}_1$ and $\mathcal{S}_2$.

```
Split D_train into K folds {D_cal^(k)}_{k=1}^K grouped by genome ID and stratified by Level 1 labels.
Initialize empty multisets S_1^(k)[y_1] and S_2^(k)[y_1] for k ∈ {1..K} and y_1 ∈ Y_1.
for k = 1 to K do
    D_prop^(k) ← D_train \ D_cal^(k)
    Train model f_k on contigs generated from D_prop^(k).
    for each genome g ∈ D_cal^(k) with true labels (y_1^*, y_2^*) do
        Extract contigs X_g using window L and stride S (optionally subsampled).
        for each contig x ∈ X_g do
            Obtain logits z = f_k(x) and probabilities p̂ = softmax(z).
            Level 1 Score: s_1 = 1 − Σ_{c∈C(y_1^*)} p̂_c
            Add s_1 to S_1^(k)[y_1^*].
            Level 2 Score (Logit Margin):
            s_2 = max_{c∈C(y_1^*)\{y_2^*}} z_c − z_{y_2^*}
            Add s_2 to S_2^(k)[y_1^*].
    Sort all multisets in S_1^(k) and S_2^(k) in ascending order for fast querying.
return {f_k}_{k=1}^K, S_1, S_2
```

Algorithm S2. Hierarchical Prediction for a New Contig using Pooled p-values

Require: Test contig x^*, models $\{f_k\}_{k=1}^K$, score banks $\mathcal{S}_1, \mathcal{S}_2$, target error rate α_{total}.
Ensure: Prediction sets Γ_1 (Level 1) and Γ_2 (Level 2).

$\alpha_1 \leftarrow \alpha_{\text{total}}/2, \alpha_2 \leftarrow \alpha_{\text{total}}/2$ ▷ Bonferroni correction across levels
$\Gamma_1 \leftarrow \emptyset, \Gamma_2 \leftarrow \emptyset$
// Level 1 Prediction
for each $y_1 \in \{\text{Metazoa}, \text{Protist}\}$ **do**
  Calculate pooled p-value:

$$p_{\text{val}}(y_1) = \frac{1 + \sum_{k=1}^K \sum_{s \in \mathcal{S}_1^{(k)}[y_1]} \mathbb{I}[s \geq s_{1,k}^*]}{1 + \sum_{k=1}^K |\mathcal{S}_1^{(k)}[y_1]|}$$

  where $s_{1,k}^* = 1 - \sum_{c \in \mathcal{C}(y_1)} \hat{p}_{k,c}(x^*)$.
  if $p_{\text{val}}(y_1) > \alpha_1$ **then**
    $\Gamma_1 \leftarrow \Gamma_1 \cup \{y_1\}$
// Level 2 Prediction
for each $y_1 \in \Gamma_1$ **do**
  Initialize $\Gamma_2[y_1] \leftarrow \emptyset$
  for each $y_2 \in \mathcal{C}(y_1)$ **do**
    Calculate pooled p-value using Logit Margin:

$$p_{\text{val}}(y_2) = \frac{1 + \sum_{k=1}^K \sum_{s \in \mathcal{S}_2^{(k)}[y_1]} \mathbb{I}[s \geq s_{2,k}^*]}{1 + \sum_{k=1}^K |\mathcal{S}_2^{(k)}[y_1]|}$$

    where $s_{2,k}^* = \max_{c \in \mathcal{C}(y_1) \setminus \{y_2\}} z_{k,c}(x^*) - z_{k,y_2}(x^*)$.
    if $p_{\text{val}}(y_2) > \alpha_2$ **then**
      $\Gamma_2[y_1] \leftarrow \Gamma_2[y_1] \cup \{y_2\}$
return Γ_1, Γ_2

Outer Evaluation (LOSO): To rigorously evaluate the model's generalization to novel viral genomes, we wrapped the aforementioned procedures in a Leave-One-Species-Out (LOSO) cross-validation scheme. For each species genome in the dataset, we held it out as the test set, applied Algorithm 1 to the remaining genomes to obtain the calibration sets and models, and then evaluated the empirical coverage and average prediction set size on the contigs of the held-out genome using Algorithm 2.

5. ALTERNATIVE GRAPH NEURAL NETWORK ARCHITECTURES

A. Graph Construction based on GVOG Sharing

To construct the viral genome network, we adapted the protein cluster sharing methodology originally proposed by vConTACT2, replacing generic protein clusters with pre-aligned Giant Virus Orthologous Groups (GVOGs). The genome-to-GVOG relationships were initially parsed into a binary incidence matrix.

We then quantified the genomic similarity between each pair of viral contigs by calculating the probability of observing at least c shared GVOGs by chance. This was modeled using a one-tailed hypergeometric distribution. For any two genomes A and B, the probability P is defined as:

$$P(X \geq c) = \sum_{i=c}^{\min(a,b)} \frac{\binom{a}{i}\binom{n-a}{b-i}}{\binom{n}{b}}$$

where:

- c is the observed number of shared GVOGs between genome A and genome B;
- a and b are the total numbers of distinct GVOGs present in genome A and genome B, respectively;
- n is the total number of distinct GVOGs across the entire dataset;
- $\binom{x}{y}$ denotes the binomial coefficient (number of combinations of x items taken y at a time).

To establish a robust threshold for edge creation, a similarity score $S_{A,B}$ was calculated by taking the negative base-10 logarithm of the hypergeometric P-value, adjusted by the total number of possible pairwise genome comparisons (Bonferroni-like correction):

$$S_{A,B} = -\log_{10}\left(P(X \geq c) \times \frac{N(N-1)}{2}\right)$$

where N is the total number of viral genomes in the dataset. Following established protocols, an edge was constructed between genome A and genome B if and only if $S_{A,B} \geq 1$. Previous permutation tests (randomly rearranging GVOGs) have demonstrated that similarity scores above this threshold do not occur by chance, ensuring the biological significance of the network topology.

B. Inductive Node Representation Learning via GraphSAGE

In the context of metagenomic viral host prediction, the model must routinely process novel, unseen viral contigs during the inference phase. Traditional Graph Convolutional Networks (GCNs) are inherently *transductive*, meaning they require the entire graph structure (including test nodes) to be present during training to learn fixed node embeddings. This makes standard GCNs unsuitable for our dynamic, real-world application.

To address this, we evaluated GraphSAGE (Graph Sample and Aggregate) [1], an *inductive* framework that learns a function to generate embeddings by sampling and aggregating features from a node's local neighborhood, rather than training individual embeddings for each node.

At the l-th layer of the GraphSAGE model, the representation for a given node v is updated through the following two-step process:

1. **Aggregation:** The representations of node v's sampled neighbors, denoted as $\mathcal{N}(v)$, are aggregated into a single vector:

$$h_{\mathcal{N}(v)}^{(l)} = \text{AGGREGATE}^{(l)}\left(\left\{h_u^{(l-1)}, \forall u \in \mathcal{N}(v)\right\}\right)$$

2. **Update:** The aggregated neighborhood vector is concatenated with the node's own representation from the previous layer, transformed by a trainable weight matrix $W^{(l)}$, and passed through a non-linear activation function σ (e.g., ReLU):

$$h_v^{(l)} = \sigma\left(W^{(l)} \cdot \text{CONCAT}\left(h_v^{(l-1)}, h_{\mathcal{N}(v)}^{(l)}\right)\right)$$

where $h_v^{(0)}$ is initialized with the sequence-derived feature vectors of the contigs. By learning the aggregator functions rather than fixed node embeddings, GraphSAGE allows GiantHost to seamlessly generalize to completely unseen viral contigs during inference, merely by observing their GVOG profiles and connecting them to the existing reference network.

6. DETAILED BENCHMARK RESULTS

In metagenomic studies, viral genomes exhibit extreme variations in length. Consequently, larger genomes naturally fragment into a disproportionately higher number of contigs compared to smaller genomes. If standard micro-averaged accuracy were applied across all contigs collectively, the evaluation metric would be heavily biased toward the performance on a few overwhelmingly large genomes. To mitigate this evaluation bias and ensure fair representation across diverse viral taxa, we introduce a *genome-resolved accuracy* metric.

Let N denote the total number of held-out viral genomes in the test set. We represent the i-th genome as G_i, where $i \in \{1, 2, \ldots, N\}$. During the evaluation process, each genome G_i is fragmented into a set of M_i contigs, denoted as $C_i = \{c_{i,1}, c_{i,2}, \ldots, c_{i,M_i}\}$.

For a given contig $c_{i,j}$, let $\hat{y}_{i,j}$ be the host prediction generated by GiantHost, and let y_i be the ground-truth host label for the parent genome G_i. We first compute the contig-level accuracy specific to the individual genome G_i, denoted as $\mathrm{Acc}(G_i)$:

$$\mathrm{Acc}(G_i) = \frac{1}{M_i} \sum_{j=1}^{M_i} \mathbb{I}(\hat{y}_{i,j} = y_i)$$

where $\mathbb{I}(\cdot)$ is the indicator function, which equals 1 if the predicted host matches the ground truth ($\hat{y}_{i,j} = y_i$), and 0 otherwise.

Finally, the overall genome-resolved accuracy is defined as the macro-average of these individual per-genome accuracies:

$$\mathrm{Acc}_{\mathrm{macro}} = \frac{1}{N} \sum_{i=1}^{N} \mathrm{Acc}(G_i)$$

By aggregating the predictions in this hierarchical manner, the metric guarantees that each viral genome contributes equally to the final performance evaluation, regardless of its original sequence length or the resulting number of fragmented contigs.

Table S1. Leave-one-genome-out accuracy across different models, feature sets, and contig lengths.

Model	Feature Set	Contig Length				
		5k	10k	15k	20k	Complete
RF	Trait	0.8519	0.8713	0.8755	0.8739	0.8716
	K-mer	0.8392	0.8519	0.8635	0.8691	0.8784
	GVOG	0.7412	0.8830	0.9048	0.9190	0.9527
	K-mer + Trait	0.8531	0.8725	0.8751	0.8838	0.8716
	GVOG + K-mer	0.8320	0.8579	0.8721	0.8777	0.9122
	GVOG + Trait	0.8537	0.8779	0.8866	0.8846	0.9324
	GVOG + K-mer + Trait	0.8467	0.8670	0.8689	0.8766	0.8986
MLP	Trait	0.8128	0.8759	0.8906	0.8960	0.9054
	K-mer	0.8618	0.8828	0.8946	0.9057	0.9122
	GVOG	0.7641	0.8953	0.9219	0.9260	0.9662
	K-mer + Trait	0.8144	0.8728	0.8896	0.8988	0.9122
	GVOG + K-mer	0.7813	0.8934	0.9249	0.9272	0.9662
	GVOG + Trait	0.8562	0.9231	0.9369	0.9455	0.9730
	GVOG + K-mer + Trait	0.8456	0.9159	0.9292	0.9368	0.9730
DualMLP	GVOG + K-mer	0.8729	0.9234	0.9378	0.9486	0.9662
	GVOG + Trait	0.8601	0.9253	0.9367	0.9409	0.9662
	GVOG + K-mer + Trait	0.8651	0.9261	0.9393	0.9400	0.9662
GNN	Trait	0.8460	0.9013	0.9150	0.9258	0.9189
	K-mer	0.8479	0.8921	0.9172	0.9237	0.9054
	K-mer + Trait	0.8465	0.8990	0.9145	0.9228	0.9189
DualGNN	GVOG + K-mer	0.8935	0.9261	0.9376	0.9381	0.9054
	GVOG + Trait	0.8682	0.9151	0.9275	0.9333	0.8919
	GVOG + K-mer + Trait	0.8729	0.9128	0.9229	0.9304	0.8986

Table S2. Leave-one-genus-out accuracy across different models, feature sets, and contig lengths.

Model	Feature Set	Contig Length				
		**5k**	**10k**	**15k**	**20k**	**Complete**
RF	Trait	0.5644	0.5938	0.6082	0.6049	0.5928
	K-mer	0.5640	0.5822	0.5957	0.6044	0.6103
	GVOG	0.5612	0.6530	0.6871	0.7063	0.8062
	K-mer + Trait	0.5785	0.6000	0.6075	0.6091	0.6105
	GVOG + K-mer	0.5696	0.6054	0.6241	0.6375	0.7127
	GVOG + Trait	0.5853	0.6237	0.6501	0.6640	0.7204
	GVOG + K-mer + Trait	0.5773	0.6088	0.6100	0.6264	0.6638
MLP	Trait	0.5056	0.5537	0.5718	0.5962	0.6373
	K-mer	0.5714	0.5809	0.5907	0.6002	0.6167
	GVOG	0.5774	0.6660	0.7147	0.7318	0.8408
	K-mer + Trait	0.4895	0.5426	0.5558	0.5821	0.5782
	GVOG + K-mer	0.5798	0.6678	0.7088	0.7294	0.8447
	GVOG + Trait	0.5530	0.6516	0.6978	0.7063	0.8639
	GVOG + K-mer + Trait	0.5560	0.6488	0.6915	0.7064	0.8495
DualMLP	GVOG + K-mer	0.6500	0.7060	0.7294	0.7553	0.8245
	GVOG + Trait	0.5950	0.6815	0.7227	0.7313	0.7995
	GVOG + K-mer + Trait	0.5971	0.6671	0.7118	0.7235	0.7745
GNN	Trait	0.5984	0.6482	0.6778	0.6986	0.6908
	K-mer	0.6180	0.6668	0.6993	0.7140	0.6689
	K-mer + Trait	0.5981	0.6496	0.6808	0.6910	0.6754
DualGNN	GVOG + K-mer	0.6488	0.6901	0.7193	0.7282	0.7337
	GVOG + Trait	0.6160	0.6671	0.7030	0.7032	0.7035
	GVOG + K-mer + Trait	0.6317	0.6831	0.7174	0.7218	0.7199

Table S3. 5-fold cross-validation accuracy across different models, feature sets, and contig lengths.

Model	Feature Set	Contig Length				
		5k	10k	15k	20k	Complete
RF	Trait	0.8456	0.8634	0.8684	0.8720	0.8651
	K-mer	0.8391	0.8616	0.8627	0.8693	0.8789
	GVOG	0.6972	0.8465	0.8789	0.8913	0.9526
	K-mer + Trait	0.8516	0.8684	0.8715	0.8703	0.8786
	GVOG + K-mer	0.8328	0.8561	0.8702	0.8793	0.9193
	GVOG + Trait	0.8475	0.8662	0.8756	0.8833	0.9195
	GVOG + K-mer + Trait	0.8501	0.8697	0.8757	0.8785	0.8989
MLP	Trait	0.7882	0.8471	0.8564	0.8565	0.8989
	K-mer	0.8356	0.8572	0.8641	0.8707	0.8920
	GVOG	0.7332	0.8585	0.8895	0.9078	0.9662
	K-mer + Trait	0.7950	0.8418	0.8477	0.8473	0.8920
	GVOG + K-mer	0.7491	0.8605	0.8913	0.9039	0.9595
	GVOG + Trait	0.8120	0.8777	0.8872	0.8956	0.9660
	GVOG + K-mer + Trait	0.8146	0.8852	0.8951	0.9045	0.9729
DualMLP	GVOG + K-mer	0.8797	0.9091	0.9178	0.9237	0.9798
	GVOG + Trait	0.8312	0.8978	0.9065	0.9063	0.9529
	GVOG + K-mer + Trait	0.8267	0.8924	0.9042	0.9072	0.9729
GNN	Trait	0.8172	0.8669	0.8822	0.8884	0.9260
	K-mer	0.8264	0.8703	0.8874	0.8962	0.9051
	K-mer + Trait	0.8196	0.8679	0.8809	0.8866	0.9055
DualGNN	GVOG + K-mer	0.8718	0.9113	0.9214	0.9299	0.8784
	GVOG + Trait	0.8394	0.8887	0.9072	0.9138	0.8853
	GVOG + K-mer + Trait	0.8435	0.8864	0.9008	0.9071	0.8989

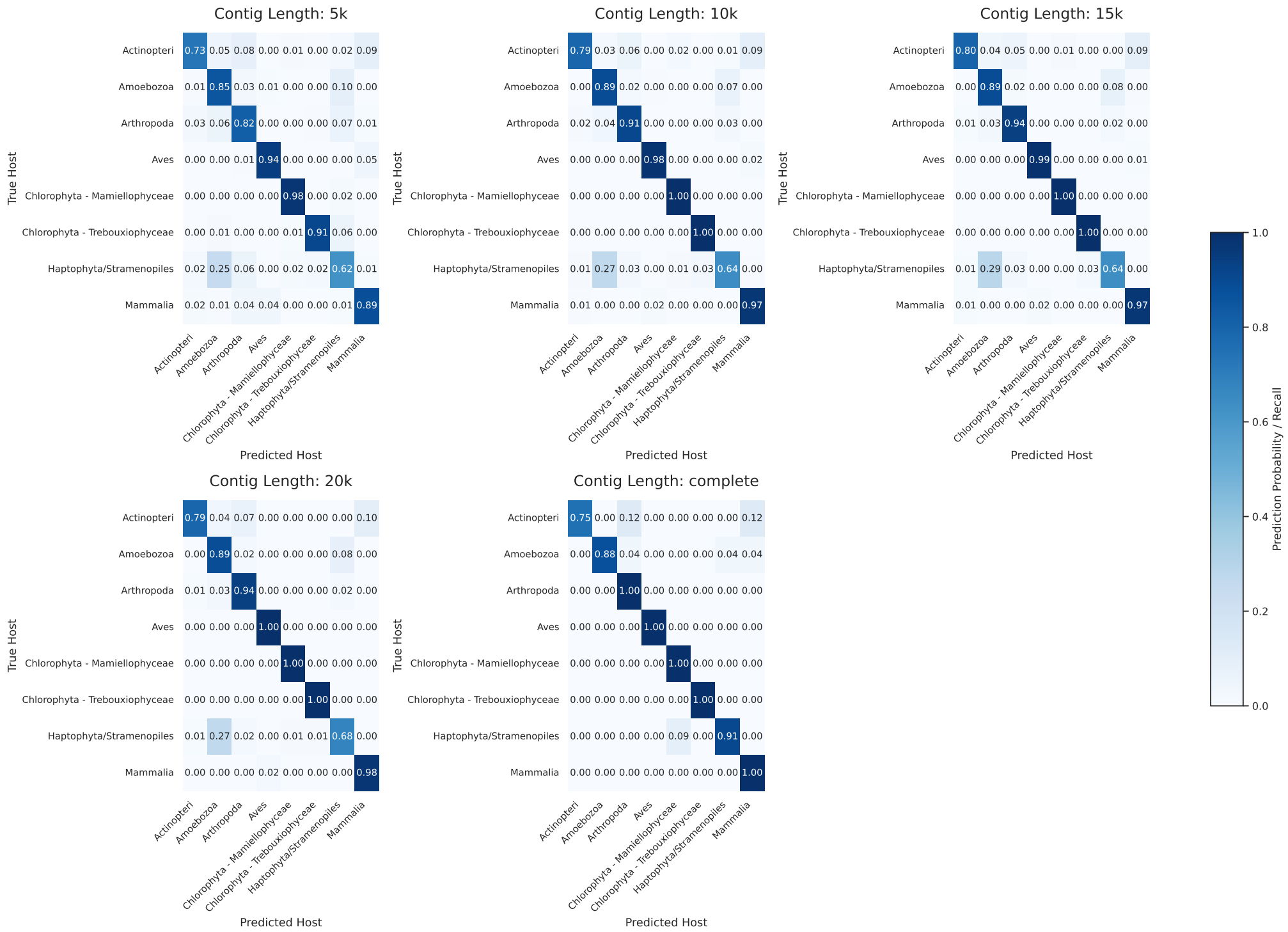

Fig. S2. The confusion matrix of GiantHost on various lengths of contigs

7. HYPERPARAMETER OF EXPERIMENTS

The random seed is 3409.

Table S4. Hyperparameter settings for all models.

Hyperparameter	MLP	DualMLP	GNN	DualGNN	Random Forest
Number of layers	1	3	1	4	–
Hidden dimension	256	256	256	256	–
Dropout rate	0.1	0.1	0.1	0.1	–
Activation function	ReLU	ReLU	ReLU	ReLU	–
Learning rate	0.001	0.001	0.001	0.01	–
Optimizer	Adam	Adam	Adam	Adam	–
Batch size	1024	1024	–	–	–
Epochs	5	5	300	200	–
Number of trees	–	–	–	–	100
Max depth	–	–	–	–	None

Table S5. Training time (seconds) and peak GPU memory usage (MB) for all model–feature combinations. Memory marked with † denotes CPU-only (Random Forest).

	MLP		DualMLP		GNN		DualGNN		Random Forest	
**Features**	Time	Mem	Time	Mem	Time	Mem	Time	Mem	Time	Mem†
GVOG	5.71	1520.7	–	–	–	–	–	–	4.75	3029.9
KMER	1.80	45.5	–	–	6.46	603.6	–	–	1.40	66.9
TRAIT	1.72	34.7	–	–	5.51	582.9	–	–	1.60	55.7
KMER+TRAIT	1.70	59.2	–	–	7.37	805.1	–	–	1.83	109.5
GVOG+KMER	6.53	1564.9	6.29	1543.9	–	–	13.75	2030.5	3.11	4630.7
GVOG+TRAIT	6.28	1544.8	7.28	1533.8	–	–	13.84	2020.2	3.00	4590.1
GVOG+KMER+TRAIT	6.32	1589.1	7.26	1556.1	–	–	13.81	2042.9	3.13	4679.8

8. COMPARISON OF BLAST-BASED SEQUENCE HOMOLOGY SEARCH AND GIANTHOST FOR HOST PREDICTION

Table S6. Comparison of BLAST-based methods and GiantHost under cross-validation benchmarking. Overall Accuracy treats contigs without predictions as incorrect.

Eval Strategy	Method	5 kb	10 kb	15 kb	20 kb
Overall Accuracy (%)					
LOSO	Strict BLAST	22.01	17.88	14.32	13.28
	Default BLAST	74.83	78.37	80.36	81.80
	GiantHost	83.82	88.50	89.82	90.58
	Combined	84.74	88.85	90.13	90.79
LOGO	Strict BLAST	0.00	0.00	0.00	0.00
	Default BLAST	38.09	46.84	51.59	54.26
	GiantHost	58.96	66.60	68.95	69.78
	Combined	58.96	66.60	68.95	69.78

To assess the relative contributions of sequence homology and learned genomic representations for host prediction, we benchmarked BLAST-based approaches alongside GiantHost under the same cross-validation framework (Supplementary Table S6). We evaluated two BLAST configurations: a strict mode (nucleotide identity > 95% and query coverage > 70%), representing the high-confidence filtering used in practice, and a default mode (best hit with evalue threshold 1e-3), representing the upper bound of homology-based prediction. Under the leave-one-species-out (LOSO) evaluation, strict BLAST achieved perfect accuracy on covered contigs but could only assign predictions to 13–22% of test sequences, yielding overall accuracies of 13.28–22.01% across fragment lengths. Default BLAST increased coverage to 75–93% with near-perfect accuracy (98.98–100%), yet this setting is methodologically problematic for real-world applications because it assigns host labels based on potentially distant homologs without quality filtering. Critically, under the leave-one-genus-out (LOGO) evaluation—the most realistic scenario for novel virus discovery—strict BLAST achieved zero coverage across all fragment lengths, demonstrating that sequence homology search is entirely unable to predict hosts for viruses from previously unseen genera. Even default BLAST only covered 38.78–56.49% of contigs in this setting, with accuracy declining to 85.71–98.22%. In contrast, GiantHost provided predictions for all input sequences regardless of their taxonomic novelty, achieving overall accuracies of 83.82–90.58% (LOSO) and 58.96–69.78% (LOGO) on fragmented contigs (5–20 kb)—consistently surpassing default BLAST in overall accuracy across all fragment lengths and evaluation strategies on fragmented contigs. These results demonstrate that GiantHost captures generalizable host-association signals beyond simple sequence homology, and its value is most pronounced for novel viruses that lack close relatives in reference databases, which is precisely the scenario encountered in metagenomic discovery of NCLDVs.

We further evaluated model performance on the subset of test contigs that received no significant BLASTn hit (E-value < 1e-5) against the training sequences, i.e., contigs for which sequence-similarity-based methods would be entirely uninformative. This "BLAST-unassignable" subset comprised 20–25% of all test contigs (e.g., 987 of 4,826 at 10 kb). Our MultiTaskMLP model still achieved 51–57% order-level accuracy on these difficult contigs (shown in Figure S3), confirming that the learned representations capture biological signals beyond sequence homology.

Also, Trait + GVOG combination shows superior performance compared to kmer+GVOG.

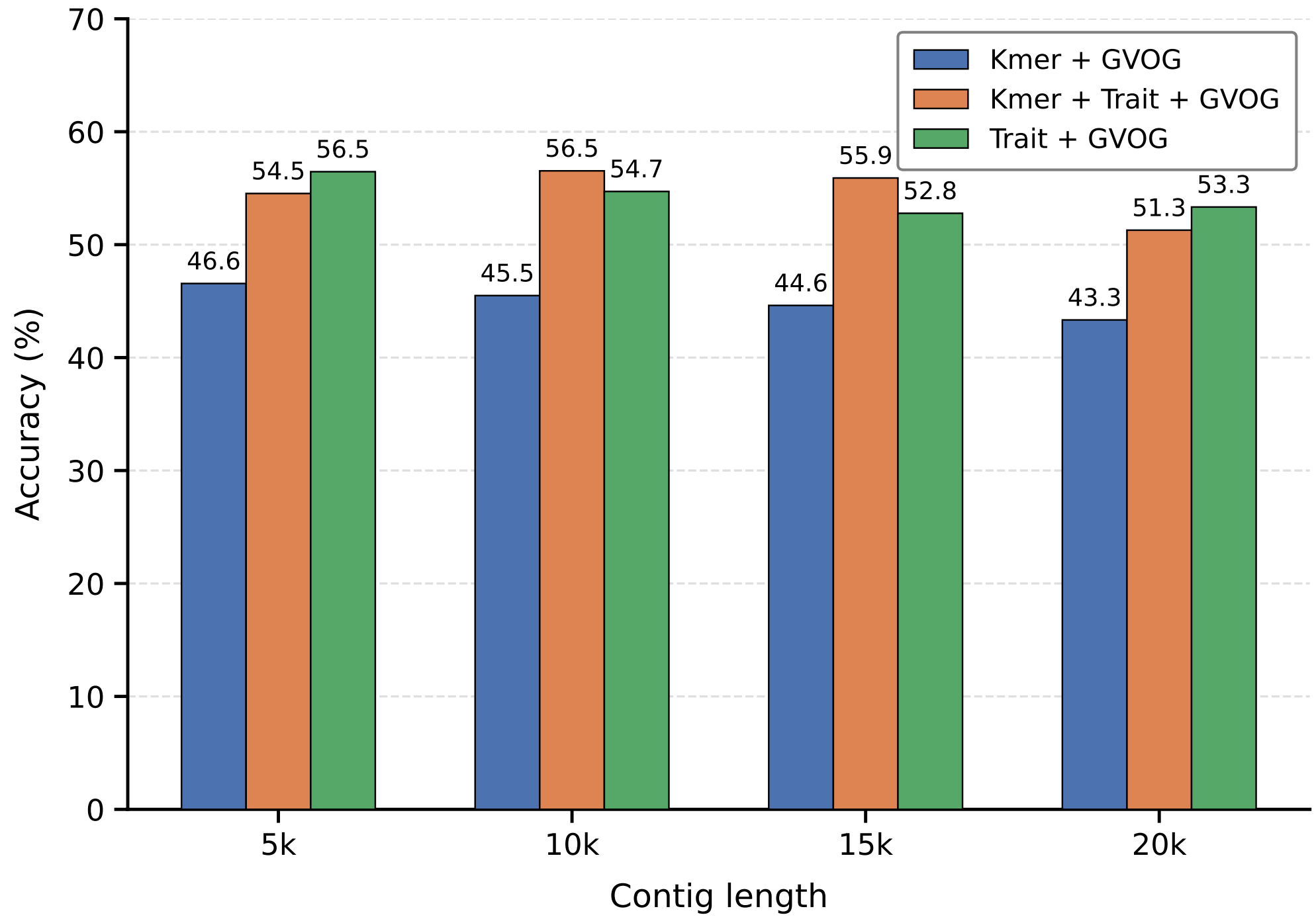

Fig. S3. Performance on BLAST-unresolvable contigs

Considering the computational cost and performance, we choose Trait+GVOG as the final setting.

9. RATIONALE AND ADVANTAGES OF THE MULTI-TASK LEARNING FRAMEWORK

In our study, we observed a slight performance drop in the primary host prediction task on the RefSeq dataset when employing the multi-task learning (MTL) framework compared to the single-task model (as shown in Figure S4). However, the adoption of the MTL architecture is a deliberate design choice aimed at maximizing the model's generalization capabilities on real-world metagenomic data. The rationale is grounded in the following three aspects. First, the RefSeq database predominantly consists of cultivated viruses, which exhibit a distinct feature distribution compared to uncultivated viruses found in environmental metagenomes (domain shift). A single-task model optimized solely for RefSeq tends to overfit the specific biases of cultivated genomes. By introducing auxiliary tasks, the MTL framework acts as a powerful regularizer, penalizing the model for memorizing dataset-specific artifacts and forcing it to learn more universal representations. Second, virus-host interactions are not isolated events but are deeply intertwined with the evolutionary trajectories of both the virus and the host. The auxiliary tasks in our MTL framework (e.g., predicting virus taxonomy and broader host groups) explicitly model this biological synergy. We hypothesize that sharing latent representations across related tasks encourages the extraction of features that capture biological signal rather than dataset-specific artifacts.

In conclusion, the marginal accuracy reduction on the RefSeq dataset is an expected and acceptable trade-off. We sacrificed localized performance on a biased dataset to achieve a more robust, biologically sound, and generalizable tool for global metagenomic exploration.

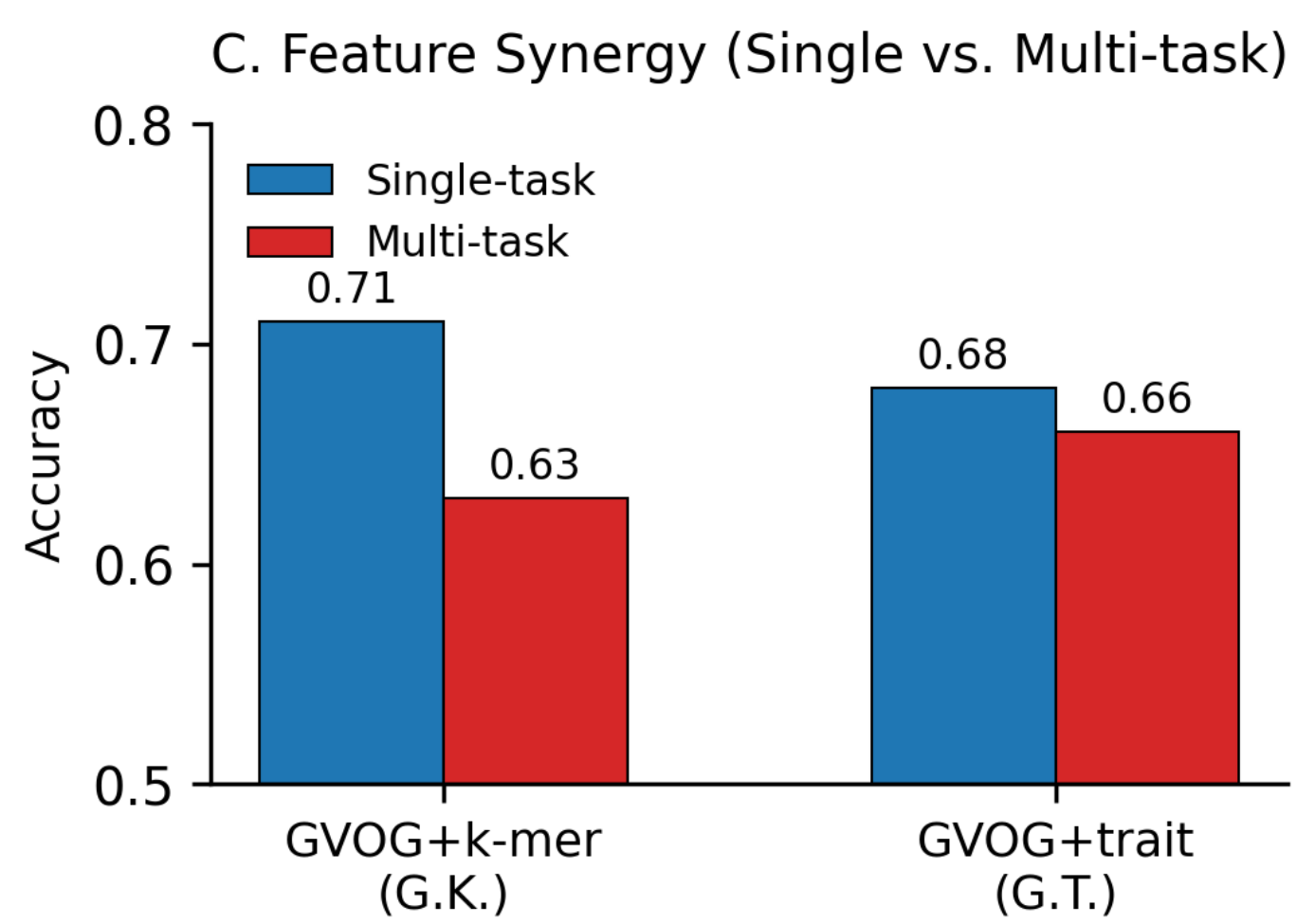

Fig. S4. Comparison of feature performance between single-task and multi-task frameworks under the leave-one-genus-out. The lower accuracy of the multi-task model is an expected trade-off to prevent overfitting to RefSeq's cultivated viruses, thereby ensuring robust generalization on real-world metagenomic data.

To demostrate the effect of multitask learning and DANN, we have conducted another ablation study. We compiled 14,652 labeled contigs spanning six NCLDV orders and 39 families, derived from metagenome-assembled genomes (MAGs). For each order containing multiple annotated families, we held out one or more complete families as the test set, targeting an overall test fraction of approximately 10%. No MAG was split across partitions, preventing information leakage from co-assembled contigs. The final split yielded 13,121 training and 1,531 test contigs, with complete family-level separation achieved in five of six orders. This setup ensures that every test contig belongs to a family entirely absent from training, making the task analogous to classifying a newly discovered viral lineage into its correct order.

We evaluated three model configurations on this benchmark. A baseline model trained solely on RefSeq reference genomes achieved an accuracy of 42%, indicating that supervised features learned from isolated reference genomes transfer poorly to metagenomic contigs from unseen families—likely due to both the domain gap between RefSeq and environmental sequences and the model's reliance on family-specific motifs absent from the held-out test families. Incorporating

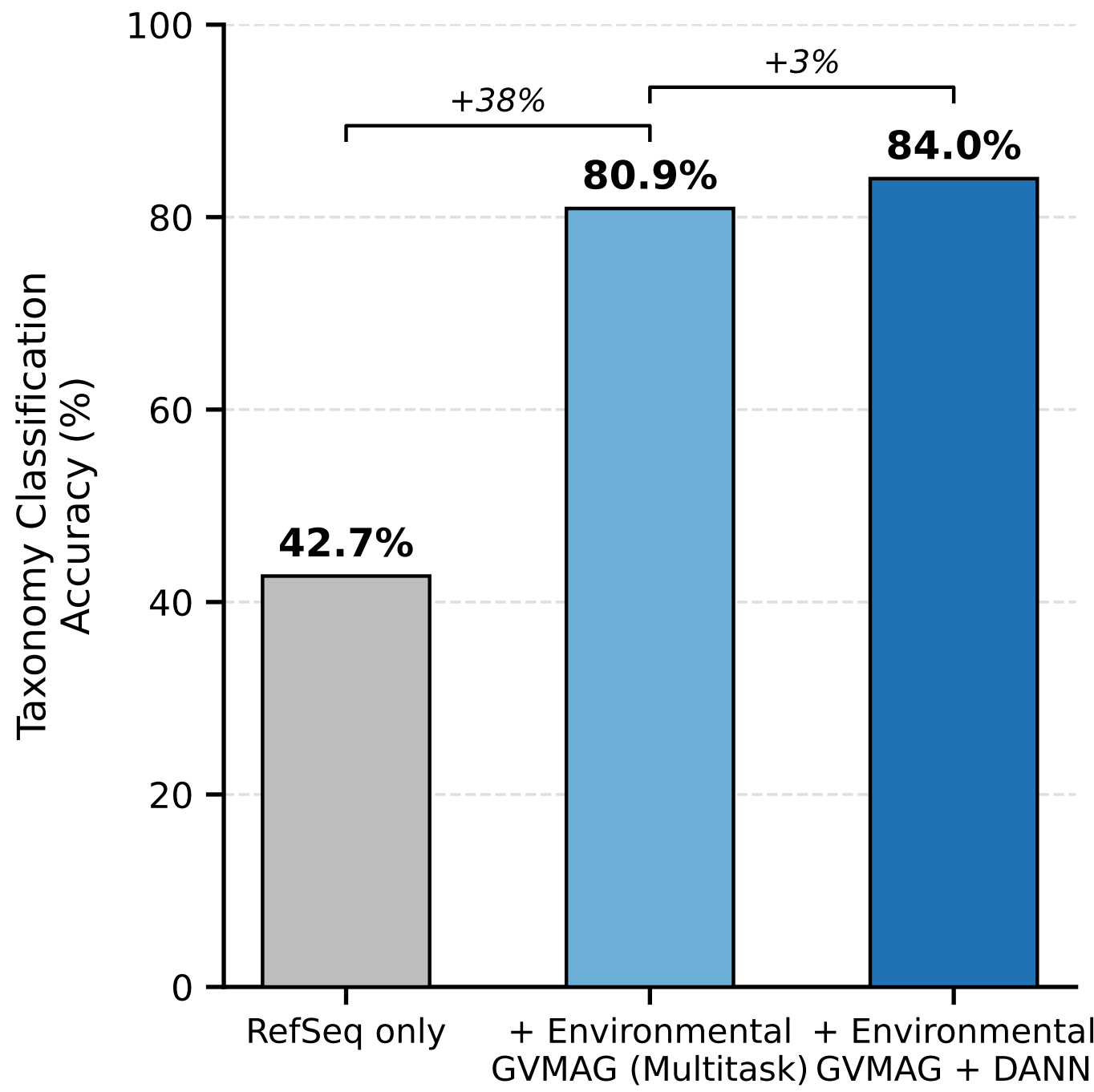

Fig. S5. .Order-level classification accuracy under the family-held-out evaluation setting.

multitask learning raised accuracy to 80%, a substantial improvement that demonstrates the auxiliary objectives encourage the encoder to capture higher-order genomic signatures shared across families within each order, rather than overfitting to family-level patterns. Finally, adding domain-adversarial training (DANN) further improved accuracy to 84%, suggesting that explicitly aligning feature distributions between source and target domains helps the model bridge residual distributional differences between training and test families, yielding more robust order-level representations.

10. ANALYSIS OF CONFORMAL PREDICTION

To comprehensively evaluate the behavior of our conformal prediction framework, we report class-wise metrics at significance level $\alpha = 0.1$ in Supplementary Table S7. Across all host classes, the empirical coverage meets or exceeds the nominal $1 - \alpha = 0.9$ guarantee, confirming the validity of the conformal calibration. No empty prediction sets (abstentions) were observed at this significance level. Notably, classes such as Aves and Chlorophyta – Mamiellophyceae achieve singleton rates above 0.9, indicating that the model can provide unique, high-confidence predictions for these well-separated host groups. In contrast, Haptophyta/Stramenopiles exhibits a lower singleton rate (0.334) and a larger average set size (3.385), reflecting greater overlap with other classes in the embedding space and thus higher intrinsic uncertainty.

Table S7. Class-wise conformal prediction metrics on the held-out test set at significance level $\alpha = 0.1$. Coverage indicates the proportion of samples whose true label is contained in the prediction set. Singleton rate reflects the proportion of samples receiving a unique prediction.

Class	Coverage	Singleton Rate	Avg Set Size	Abstention Rate	*N*
Actinopteri	0.860	0.588	2.098	0.000	967
Amoebozoa	0.978	0.738	1.623	0.000	8832
Arthropoda	0.930	0.615	2.152	0.000	2201
Aves	1.000	0.914	1.137	0.000	708
Chlorophyta – Mamiellophyceae	0.995	0.939	1.094	0.000	1091
Chlorophyta – Trebouxiophyceae	0.994	0.787	1.307	0.000	1233
Haptophyta/Stramenopiles	0.950	0.334	3.385	0.000	2129
Mammalia	0.988	0.559	1.630	0.000	3113

To assess the sensitivity of these metrics to the choice of significance level, we varied α across {0.01, 0.05, 0.1, 0.15, 0.2, 0.25, 0.3, 0.35, 0.4, 0.45, 0.5} and report the resulting overall coverage, singleton rate, average prediction set size, and abstention rate in Supplementary Figure S6. As expected, increasing α relaxes the coverage requirement, leading to higher singleton rates and smaller prediction sets at the cost of reduced coverage guarantees. This trade-off allows practitioners to select an operating point that balances prediction specificity against reliability depending on their application needs.

To investigate whether the uncertainty generated by the conformal prediction framework is biologically informative, we analyzed the composition of prediction sets containing exactly two labels. As shown in Supplementary Figure S7, the most frequently confused label pairs are not random but share deep taxonomic or ecological relationships.

For instance, the second most frequent pair, Aves and Mammalia, are both warm-blooded vertebrates (amniotes) sharing highly similar viral families. Similarly, the model frequently outputs sets containing two different classes of green algae (Chlorophyta – Mamiellophyceae and Chlorophyta – Trebouxiophyceae), highlighting its ability to narrow down the host to a specific phylum even when class-level resolution is uncertain. Furthermore, the top pair (Amoebozoa & Haptophyta/Stramenopiles) represents microbial eukaryotes that frequently co-occur in similar aquatic environments, suggesting that the model might also capture ecological signatures embedded in viral sequences. Together, these results confirm that the model's uncertainty is bounded within biologically meaningful contexts.

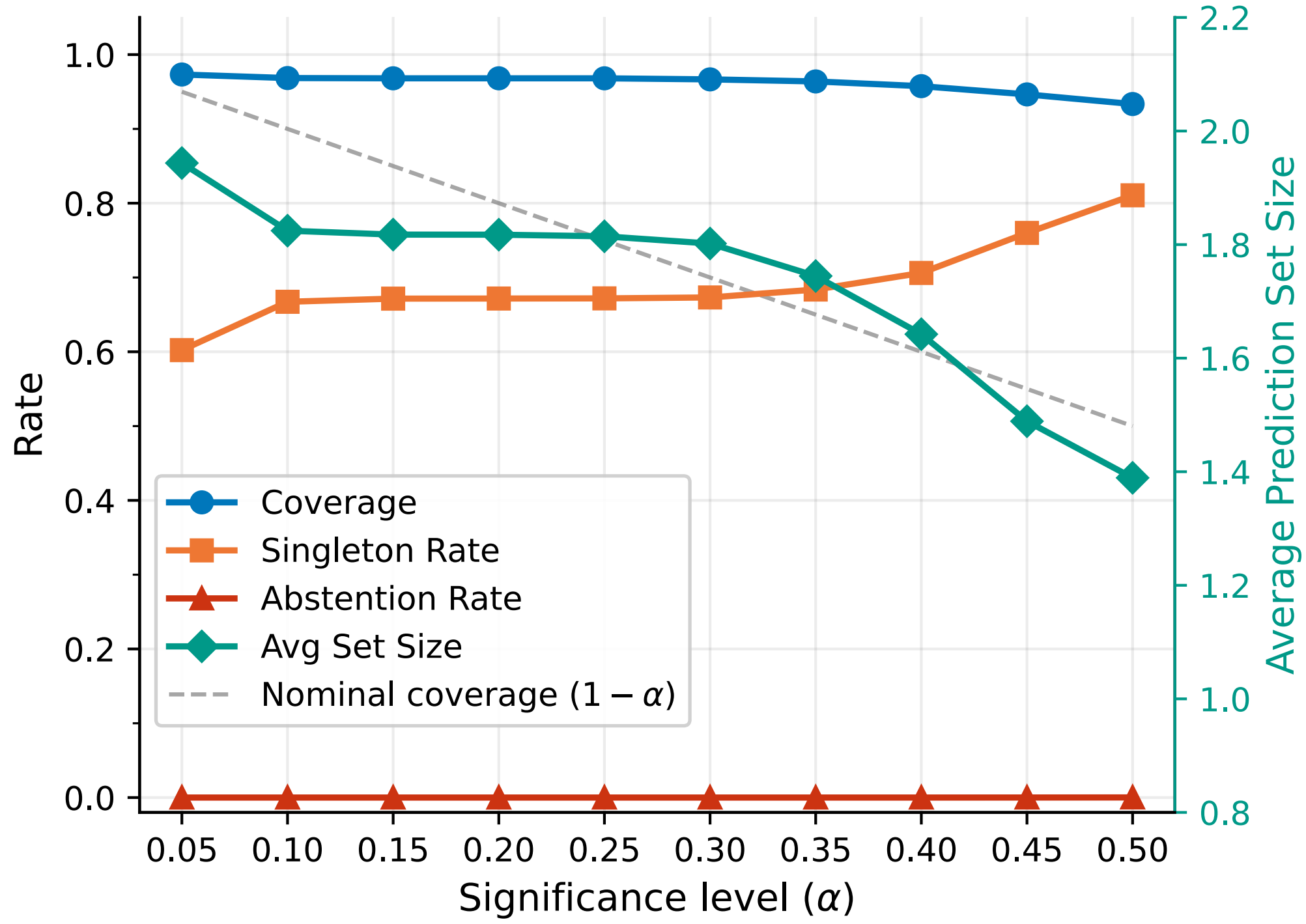

Fig. S6. Effect of significance level α on overall coverage, singleton rate, average prediction set size, and abstention rate.

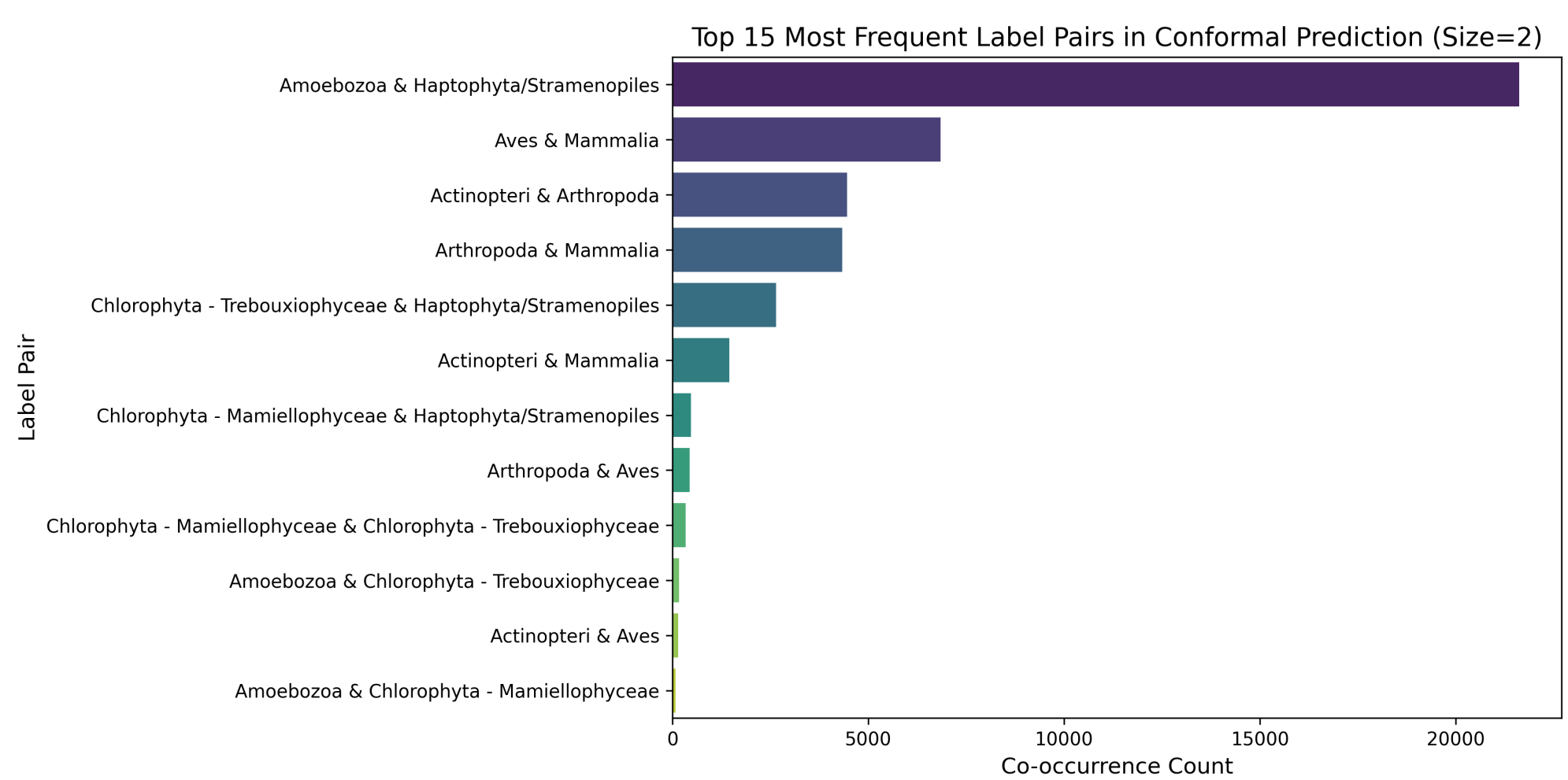

Fig. S7. The bar chart displays the most common combinations of host labels when the model outputs exactly two predictions. The high frequency of taxonomically related pairs (e.g., Aves & Mammalia, or classes within Chlorophyta) and ecologically co-occurring pairs (e.g., Amoebozoa & Haptophyta/Stramenopiles) demonstrates that the model's uncertainty reflects inherent biological ambiguity rather than random noise.

11. IMPACT OF DOMAIN-ADVERSARIAL NEURAL NETWORK

To quantitatively assess the impact of our Domain-Adversarial Neural Network (DANN) module in this bridging process, we analyzed the pairwise cosine similarities of the GVMAG embeddings before and after adversarial training (Figure S8). Before introducing DANN, the similarity distribution exhibited a sharp peak near 0.95. This indicates a severe domain collapse: the model predominantly recognized the "source domain" signature (e.g., sequencing or assembly biases inherent to GVMAGs) rather than underlying biological traits, forcing GVMAGs to cluster tightly together. Following the implementation of DANN with the gradient reversal layer, the similarity distribution significantly flattened and shifted leftward, revealing a bimodal distribution with a new peak around 0.6. This increased variance demonstrates that the adversarial training successfully stripped away the domain-specific artifacts. Consequently, the model was forced to learn domain-invariant, host-relevant features, causing the GVMAG embeddings to disperse across the latent space. This dispersion is exactly what enables the GVMAGs to align with both RefSeq and environmental contigs, effectively mitigating domain bias and ensuring that GiantHost's predictions on novel environmental data are driven by genuine biological signals rather than dataset-specific artifacts.

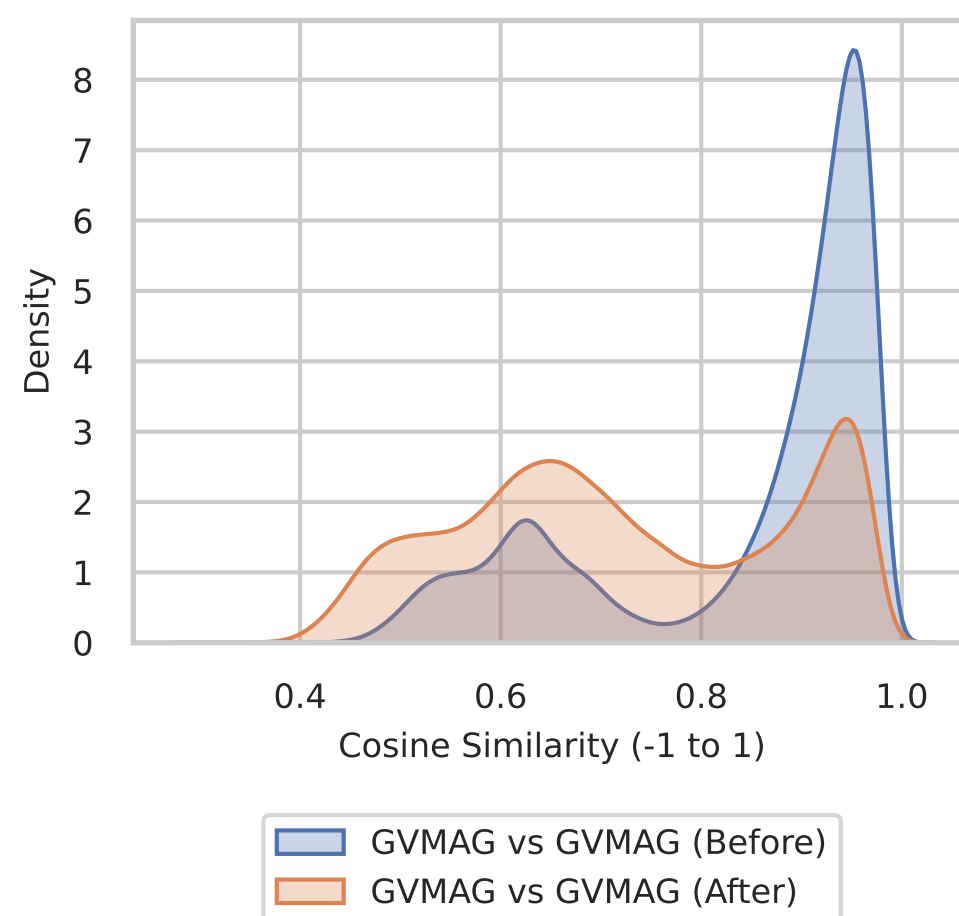

Fig. S8. Kernel Density Estimation (KDE) of pairwise cosine similarities among GVMAG embeddings. The plot compares the similarity distributions before (blue) and after (orange) the application of Domain-Adversarial Neural Network (DANN) training. Prior to DANN, GVMAG embeddings were highly similar to each other, indicating a strong domain-specific bias. After adversarial training, the distribution flattens and shifts towards lower similarity values, demonstrating that domain-specific signatures are suppressed, allowing GVMAGs to disperse and align with the broader biological feature space of RefSeq and environmental contigs.

12. APPLICATION OF GIANTHOST TO REAL METAGENOMIC DATA

Raw metagenomic sequencing reads were first subjected to rigorous quality control using fastp [2] to remove adapter sequences, low-quality bases, and excessively short reads. To maximize the recovery of contigs and capture a broader diversity of viral and host genomes, high-quality clean reads from multiple samples collected at the same sampling site were pooled together and co-assembled using MEGAHIT [3]. Subsequently, to quantify the relative abundance of the assembled contigs across different samples, the clean reads from each individual sample were mapped back to the co-assembled contigs using Bowtie2 [4]. The resulting alignment files were then converted, sorted, and indexed into BAM format using SAMtools [5]. Finally, the abundance of each contig was calculated and normalized as Transcripts Per Million (TPM) using CoverM [6] based on the sorted BAM files. This standardized TPM metric was utilized for all downstream ecological profiling and host-virus interaction analyses.

To further investigate the ecological distribution of Nucleocytoplasmic Large DNA Viruses (NCLDVs) in natural freshwater ecosystems, we applied our analytical pipeline to metagenomic datasets collected from five distinct sampling sites along the Yangtze River: Chongqing, Fuling, Jiujiang, Nanjing, and Sanxia (Three Gorges). As illustrated in Figure S9, the relative abundance of identified NCLDVs (calculated as the ratio of NCLDV sequences to the total metagenomic sequences) exhibited substantial spatial variation across the sampling locations. The highest proportion of NCLDVs was observed in the upstream region, specifically in Chongqing (0.040), closely followed by the Sanxia reservoir area (0.035). In contrast, the middle and lower reaches of the river demonstrated a notably lower NCLDV prevalence, with the ratio dropping to 0.006 in Jiujiang and 0.012 in Nanjing. This pronounced spatial heterogeneity suggests that the distribution and proliferation of giant viruses in the Yangtze River are highly dynamic, potentially driven by local environmental gradients, hydrological conditions, or the availability of specific eukaryotic hosts in different river sections.

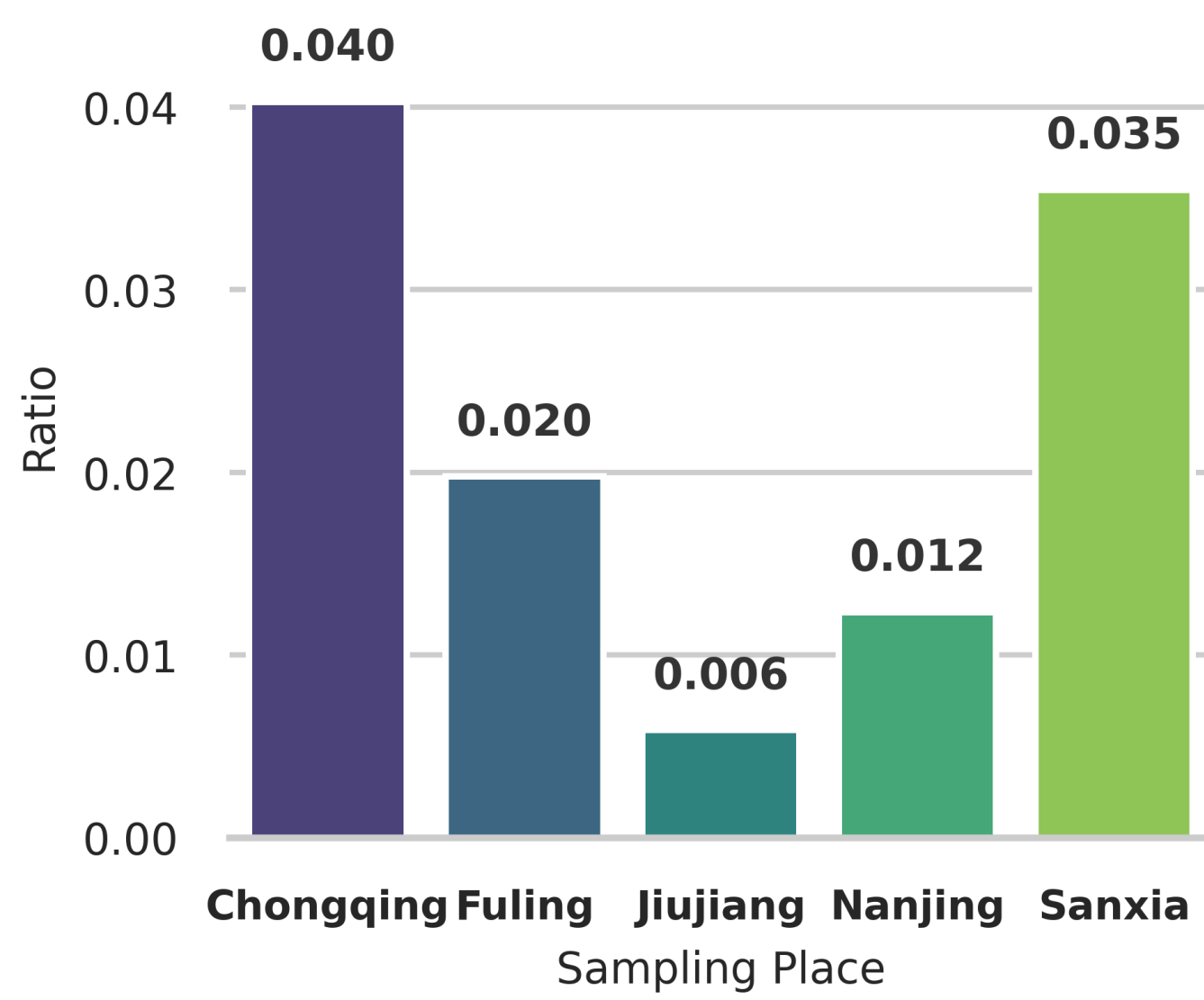

Fig. S9. Spatial distribution of NCLDV relative abundance across the Yangtze River. The bar chart illustrates the ratio of identified NCLDV sequences to the total metagenomic sequences at five distinct sampling sites (Chongqing, Fuling, Jiujiang, Nanjing, and Sanxia). The values above each bar indicate the exact calculated ratio for the corresponding location, highlighting the spatial heterogeneity of giant virus distribution in the freshwater ecosystem.

To further elucidate the ecological roles and potential virus-host interactions of the identified NCLDVs, we predicted their putative eukaryotic hosts across the five sampling sites. As depicted in Figure S10, the relative abundance of predicted host taxa reveals a distinct spatial pattern. In the majority of the sampling locations (Chongqing, Fuling, Sanxia, and Nanjing), the viral communities were predominantly associated with *Amoebozoa* and the H/S group, which is

consistent with the well-documented role of amoebae as major natural reservoirs for giant viruses in aquatic ecosystems. However, a striking anomaly was observed at the Jiujiang sampling site. Here, the relative abundance of *Amoebozoa*-associated NCLDVs experienced a sharp decline, while the proportion of viruses predicted to infect *Mammalia* exhibited a dramatic surge, accounting for nearly half of the host profile. Given that the Jiujiang sampling site is situated adjacent to agricultural farmland, this significant shift in the host spectrum strongly suggests anthropogenic impacts or agricultural runoff. The elevated mammalian viral signals are likely indicative of localized contamination from livestock or other agricultural activities. This finding highlights the sensitivity of NCLDV community structures to local environmental disturbances and underscores their potential utility as biological indicators for terrestrial runoff and anthropogenic pollution in riverine ecosystems.

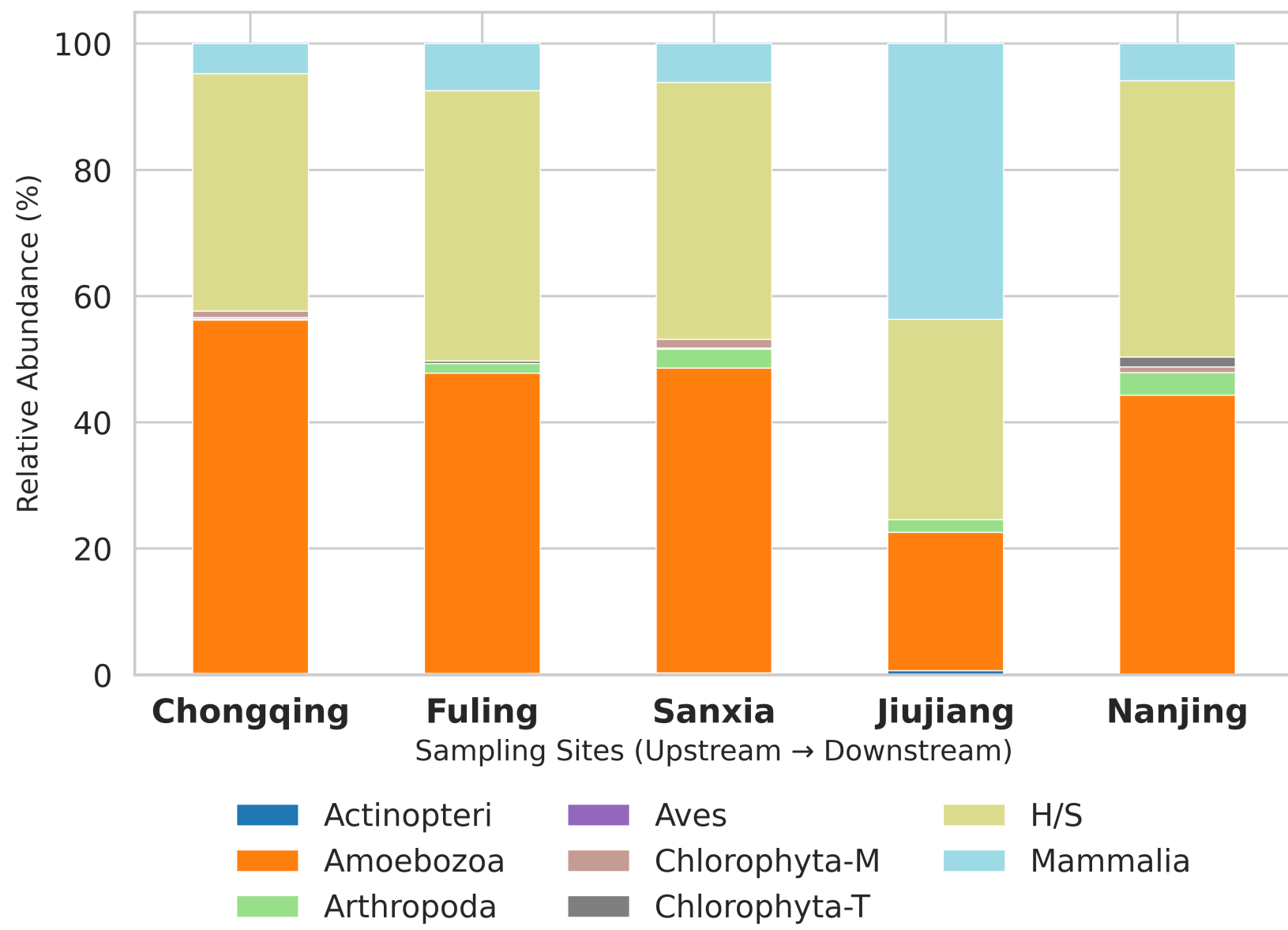

Fig. S10. Predicted host distribution of NCLDVs across the Yangtze River sampling sites. The stacked bar chart displays the relative abundance (%) of putative host taxa for the identified NCLDVs at each location, ordered from upstream to downstream. While *Amoebozoa* represents the dominant host group in most regions, the Jiujiang site exhibits a distinct profile characterized by a substantial increase in *Mammalia*-associated viruses, likely reflecting localized agricultural or anthropogenic inputs.

REFERENCES

1. W. Hamilton, Z. Ying, and J. Leskovec, "Inductive representation learning on large graphs," Adv. neural information processing systems **30** (2017).
2. S. Chen, "fastp 1.0: An ultra-fast all-round tool for fastq data quality control and preprocessing," Imeta **4**, e70078 (2025).
3. D. Li, C.-M. Liu, R. Luo, *et al.*, "Megahit: an ultra-fast single-node solution for large and complex metagenomics assembly via succinct de bruijn graph," Bioinformatics **31**, 1674–1676 (2015).
4. B. Langmead, C. Wilks, V. Antonescu, and R. Charles, "Scaling read aligners to hundreds of threads on general-purpose processors," Bioinformatics **35**, 421–432 (2019).
5. P. Danecek, J. K. Bonfield, J. Liddle, *et al.*, "Twelve years of SAMtools and BCFtools," GigaScience **10** (2021). Giab008.
6. S. T. Aroney, R. J. Newell, J. N. Nissen, *et al.*, "Coverm: read alignment statistics for metagenomics," Bioinformatics **41**, btaf147 (2025).
